# Further elaboration of the stereodivergent approach to chaetominine-type alkaloids: synthesis of the reported structures of aspera chaetominines A and B and revised structure of aspera chaetominine B

**DOI:** 10.3762/bjoc.21.162

**Published:** 2025-10-13

**Authors:** Jin-Fang Lü, Jiang-Feng Wu, Jian-Liang Ye, Pei-Qiang Huang

**Affiliations:** 1 Department of Chemistry, Fujian Provincial Key Laboratory of Chemical Biology, College of Chemistry and Chemical Engineering, Xiamen University, Xiamen, Fujian 361005, P. R. China

**Keywords:** epoxidation, selective epimerization, stereodivergent synthesis, structural revision, tandem reaction

## Abstract

We report herein the fourth generation of our synthetic strategy to chaetominine-type alkaloids featuring two modifications of the last step of our 4 to 6-step approach. Firstly, by employing EDCI/HOBt as the coupling system for the last step of the one-pot *O*-debenzylation–lactamization reaction, the overall yield of our previous total synthesis of (–)-isochaetominine A was increased from 25.4% to 30.8% over five steps. Secondly, a new protocol featuring the use of an aged solution of K_2_CO_3_/MeOH to quench the DMDO epoxidation-triggered cascade reaction was developed, which allowed the in situ selective mono- or double epimerization at C11/C14 as shown by the diastereodivergent synthesis of a pair of diastereomers of versiquinazoline H from its tripeptide precursor. This double epimerization at the last-step allowed the enantiodivergent synthesis of two enantiomers in either racemate form or two pure enantiomers from the same precursor. The former was demonstrated by the synthesis of alkaloid 14-*epi*-isochaetominine C that was used to determine the enantiomeric excess of the synthesized natural product (98.7% ee), while the latter was illustrated by the synthesis of both enantiomers of the alkaloid isochaetominine. Additionally, the reported structures of alkaloids aspera chaetominines A and B have been synthesized. Moreover, the four-step synthesis of the reported structure of aspera chaetominine B generated another diastereomer that was converted in one-pot to (–)-isochaetominine C, which turned out to be the revised structure of aspera chaetominine B.

## Introduction

In contemporary organic chemistry, due to the widespread application of modern separation and analytical techniques, the structural elucidation and confirmation of natural products is no longer a motivation for the total synthesis [1]. Nevertheless, we also witness that each year, cases continue to be reported on the total synthesis enabled revision of misassigned structures of natural products [1–9].

**Figure 1:**
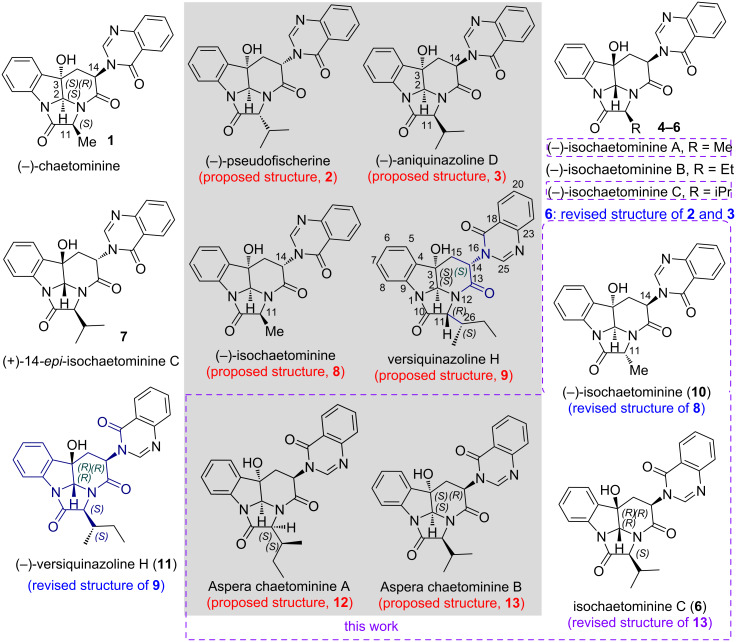
Structures of some reported chaetominine-type alkaloids and revised structures via our total syntheses.

Efficiency is one of the major concerns in the field of total synthesis of natural products [10–20], which is not only essential for organic chemistry in its own right, but also crucial for drug discovery and structural revision of natural products. Although more and more diastereomeric and enantiomeric natural products have been discovered [21–32], and divergent synthetic methodology has attracted attention in recent years [33–38], diastereodivergent and enantiodivergent total synthesis remain rare [39–54]. This is the case for (−)-chaetominine (**1** in Figure 1), which is a hexacyclic quinazolinone alkaloid possessing four stereogenic centers, first isolated from a solid-substrate culture of *Chaetomium* sp. IFB-E015 [21]. Subsequently, several homologues, diastereomers, and enantiomers of chaetominine have been reported, which include: 1) pseudofischerine (**2**) [22], isolated from the fungus *Neosartorya pseudofischeri* S. W. Peterson, and from the marine-derived fungus *Pseudallescheria boydii* F19-1 [23]; 2) aniquinazoline D (**3**), isolated from marine-derived endophytic fungus *Aspergillus nidulans* [24]*;* 3) (–)-isochaetominines A−C (**4** - **6**) and (+)-14-*epi*-isochaetominine C (**7**), isolated from the solid-substrate culture of an *Aspergillus* sp. Fungus [25], and from other sources for (–)-isochaetominine C (**6**) [26–29]; 4) isochaetominine (**8**) from *Aspergillus fumigatus*, an endophytic fungus from the liverwort *Heteroscyphus tener* (Steph.) Schiffn. [30]; (–)-versiquinazoline H (**9**), isolated from the gorgonian (*Pseudopterogorgia* sp.)-derived fungus *Aspergillus versicolor* LZD-14-1 [31]; as well as 5) aspera chaetominines A and B (**12** and **13**), isolated from marine sponge associated fungus *Aspergillus versicolour* SCSIO XWS04 F52 [32]. All these alkaloids distinguish each other only by alkyl substituent at C11 and by relative stereochemistries at C2, C3, C11, and C14.

Soon after the report of the isolation, structural elucidation, and bioactivity of (–)-chaetominine by Tan and co-workers [21], its synthesis has attracted attention of the synthetic community. The group of Snider [47], Evano [48–49], and Papeo [50] reported several elegant highly enantio- and diastereoselective total syntheses of this alkaloid from ᴅ-tryptophan. With our longstanding interest in the efficient total synthesis of natural products [10,55–56], in early 2009, our group disclosed a highly efficient four-step, enantioselective and diastereodivergent synthesis of (–)-chaetominine (**1**) and with one more step, of another diastereomer [57–58]. The strategy features a DMDO oxidation-triggered [59] double cyclization of an intermediate derived from ᴅ-tryptophan [60–61]. Subsequently, we developed a five-step total synthesis of (–)-chaetominine (**1**) and two diastereomers from ʟ-tryptophan [62]. Taking advantages of the high efficiency and flexibility of our strategy [60], we have synthesized several natural and unnatural homologues and diastereomers of chaetominines [57–58,60–65]. This allowed us to revise the proposed structures of (–)-pseudofischerine (**2**) and (–)-aniquinazoline D (**3**) both to (–)-isochaetominine C (**6**), and that of isochaetominine (**8**) to **10** (11-*epi*-chaetominine). More recently, we have communicated the revision of the structure of versiquinazoline H to **11**. During and after the latter work, we undertook further investigation on the last step of our approach to chaetominine-type alkaloids, namely, the lactamization reaction for synthesizing 3,14-*cis*-chaetominines and the DMDO epoxidation-triggered double cyclization reaction. In addition, the synthesis of the recently reported natural products aspera chaetominines A and B (**12** and **13**) [32] was addressed. Herein, we report the full accounts of these investigations, which include: 1) an improved five-step total synthesis of (–)-isochaetominine A (**4**) and a diastereomer; 2) the diastereo- or enantiodivergent syntheses of chaetominine-family alkaloids and stereoisomers, and 3) the reported structures of aspera chaetominines A and B (**12** and **13**) and revised structure of aspera chaetominine B: **6** [(–)-isochaetominine C].

## Results and Discussion

### Improved five-step total synthesis of (–)-isochaetominine A

**Scheme 1:**
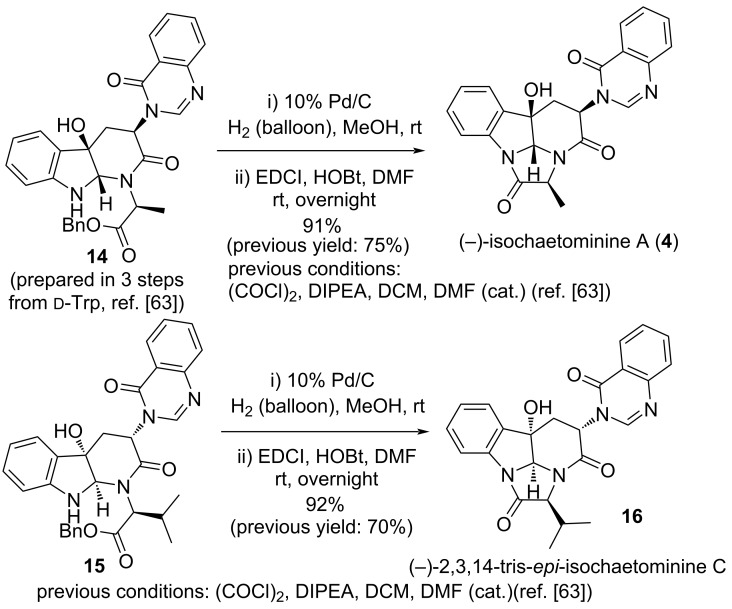
Improved total synthesis of (–)-isochaetominine A (**4**) and diastereomer **16**.

In our recent communication on the synthesis of versiquinazoline H (**11**) [65], we uncovered that the employment of EDCI/HOBt as the coupling system for the last step, namely, the *O*-debenzylation–lactamization reaction, afforded much higher yields than those using (COCl)_2_/DIPEA in our previous synthesis of isochaetominines [63]. We realized that employing this lactamization protocol would significantly improve the synthetic efficiency of isochaetominines **4**–**6**. To showcase this possibility, the improved lactamization protocol was applied to compound **14**, an intermediate in our synthesis of (–)-isochaetominine A (**4**) [63]. Indeed, EDCI/HOBt-mediated lactamization of **14** derived amino acid (not shown) via debenzylation increased the yield of (–)-isochaetominine A (**4**) from 75% to 91% (Scheme 1). Thus, overall yield of the total synthesis of (–)-isochaetominine A (**4**) increased to 30.8% over five steps. Similarly, EDCI/HOBt-mediated lactamization of amino acid (not shown) derived from **15** [63] via debenzylation produced the known (–)-2,3,14-tris-*epi*-isochaetominine C (**16**) with a significantly increased 92% yield.

### The epoxidation-triggered stereodivergent synthesis of diastereomers of versiquinazoline H: the fourth generation strategy

**Scheme 2:**
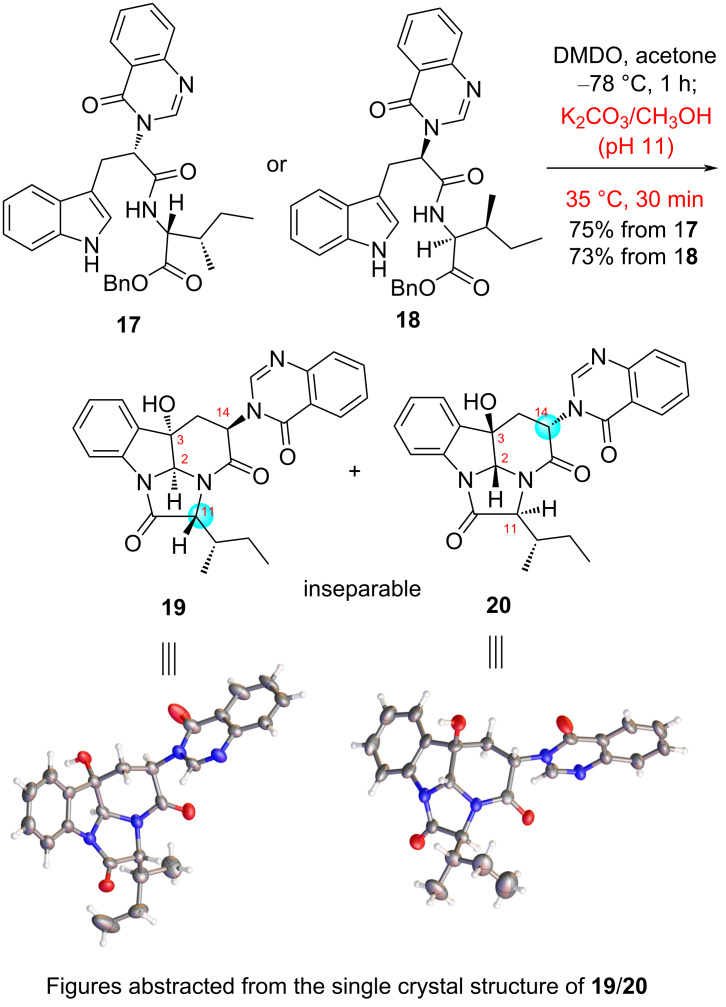
Diastereoconvergent transformations of **17** and **18** into two diastereomers of versiquinazoline H.

In all our previous syntheses of chaetominine and isochaetominine alkaloids [57–58,60–65], the key DMDO-oxidation triggered double cyclization always accompanied with a monocyclization product. It was anticipated that if we run the work-up procedure under more basic conditions, one would be able to obtain solely double cyclization products. Indeed, alternation of the work-up protocol by employing an aged solution of K_2_CO_3_/MeOH (stood at rt overnight, pH 11) to quench the DMDO oxidation reaction of intermediate **17** [65] yielded the thermodynamically stable C2/C11-*trans* and C3/C14-*trans* diastereomers **19** and **20** (dr = ca. 1:1) in a combined yield of 75% (Scheme 2). The ^1^H NMR spectrum of this diastereomeric mixture shows only one set of resonance signals, but two sets of resonance signals were observed on the ^13^C NMR spectrum. We have succeeded in preparing a single crystal from the oxidation products. Interestingly, the X-ray diffraction analysis [66] showed that the single crystal contained two diastereomers (**19**/**20**) with the structures displayed in Scheme 2. Similarly, the DMDO oxidation of diastereomers **18** followed by quenching the reaction with an aged solution of K_2_CO_3_/MeOH resulted in the formation of the same diastereomeric mixture **19** and **20** (dr = ca. 1:1) as that obtained from **17**.

### Enantiodivergent syntheses of chaetominine-type alkaloids and antipodes in racemic or enantiomeric forms

**Scheme 3:**
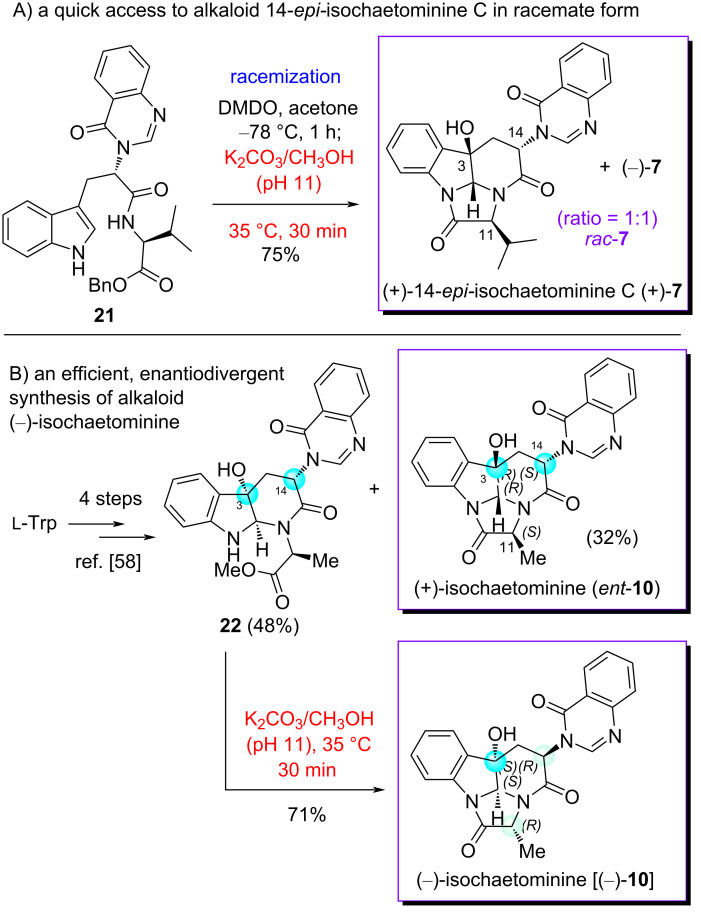
Mono- and double epimerization-based enantiodivergent syntheses of chaetominine-type alkaloids and antipodes.

Applying the newly developed quenching protocol featuring the use of an aged K_2_CO_3_/MeOH solution to the known compound **21** [63] resulted in the formation of an indistinguishable mixture of two double cyclization products (+)-14-*epi*-isochaetominine C (**7**) and its antipode (–)-**7** (ratio = ca*.* 1:1) in 75% yield (Scheme 3a). The optical rotation of this mixture is zero confirming that the reaction led to two enantiomers in almost equal quantities. To take advantage of this protocol, the enantiomeric excess (ee) of (+)-**7**, prepared in four steps from ʟ-Trp [63], was determined to be 98.7%. It is worth mentioning that in the field of chiral drug R & D, the evaluation of bio-profiles of both enantiomers and racemic compound is required by the U.S. FDA. The result presented herein shows that by a late-stage racemization, one could obtain a racemic sample in just one step, instead of repeating the total synthesis from another enantiomeric chiral starting material or racemic one. On the basis of this consideration, such racemization represents a valuable “enantiodivergent synthesis”.

Nevertheless, a more useful and generally acceptable enantiodivergent synthesis is the one that allows accessing two enantiomers both in pure form from only one enantiomer of a chiral starting material or a chiral ligand [33]. To demonstrate the value of our protocol in this regard, an enantiodivergent synthesis of isochaetominine (**10**) was envisioned. Previously, we have reported a four-step synthesis of (+)-isochaetominine (*ent*-**10**, previously known as *ent*-11-*epi*-chaetominine) in 32% yield starting from ʟ-Trp and ʟ-Ala [64]. In the last step of that total synthesis, the diastereomeric monocyclization product **22** was obtained as the major product (48% yield). Exposing this compound to an aged K_2_CO_3_/MeOH solution at 35 °C for 30 min resulted in the formation of C11/C14 double epimerization product (–)-isochaetominine [(–)-**10**] in 71% yield. Thus, we have achieved a really enantiodivergent synthesis of alkaloid (–)-isochaetominine [(–)-**10**] and its antipode (+)-**10** in just five total steps.

### Synthesis of the proposed structures of aspera chaetominines A and B and revision of stereochemistry of aspera chaetominine B

Several years after we have had accomplished the abovementioned investigations, Liu and co-workers reported the isolation and structural elucidation of two new alkaloids, aspera chaetominines A (**12**) and B (**13**) from marine sponge associated fungus *Aspergillus versicolour* SCSIO XWS04 F52 [32]. They reported that both the two alkaloids showed cytotoxic activity against leukaemia K562 and colon cancer cells SW1116 with IC_50_ ranged from 7.5 to 12.5 μM, and significant protection against H1N1 virus-induced cytopathogenicity in MDCK cells with IC_50_ values of 15.5 and 24.5 μM, respectively. Attracted by both their structure and bioactivities, we anticipated their synthesis. The author determined the structure by means of spectroscopic (^1^H, ^13^C NMR, HSQC, HMBC, and ^1^H-^1^H COSY), and MS analysis, and claimed that “their absolute configuration was unambiguously determined by the comparison with the reported compound chaetominine (**1**)” [32]. The only information regarding the absolute configuration mentioned in the text is as follows: “aspera chaetominine A (**1**) was presumably biosynthesized from ʟ-isoleucine, ʟ-valine, anthranilic acid, and ᴅ-tryptophan”. Such speculation regarding the absolute configurations is clearly not convincing. Moreover, neither optical rotation data nor melting point (both **12** and **13** were isolated as white powder) have been reported by Liu et al. [32]. Additionally, the solvents used for measuring ^1^H and ^13^C NMR are methanol-*d*_4_ that is different from Tan’s work [21] who used DMSO-*d*_6_ to record ^1^H and ^13^C NMR spectra of (–)-chaetominine (**1**).

**Scheme 4:**
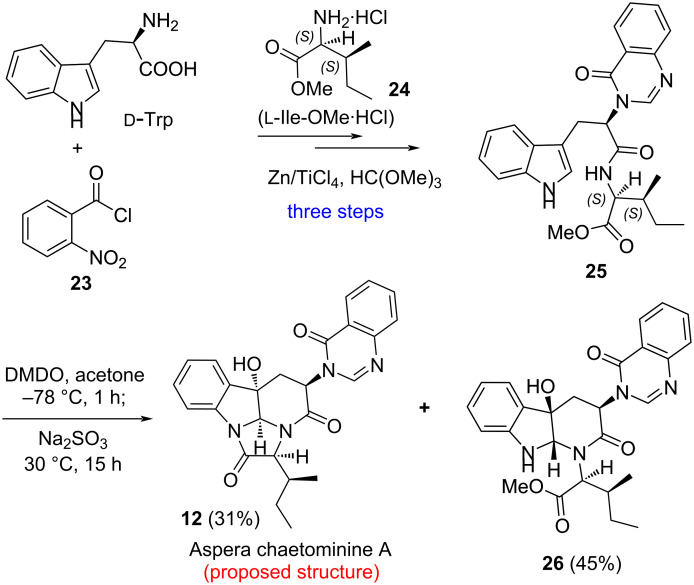
Enantioselective synthesis of the proposed structure of aspera chaetominine A.

Thus we undertook the synthesis of the proposed structures of aspera chaetominines A (**12**) and B (**13**). By adopting our first-generation strategy [57–58,61], we re-synthesized tripepetide derivative **25** from ᴅ-tryptophan (ᴅ-Trp) and ʟ-isoleucine (ʟ-Ile) methyl ester hydrochloride salt (**24**) in three steps (Scheme 4). Treating **25** with DMDO followed by work-up with a saturated aqueous solution of Na_2_SO_3_ at 30 °C provided the proposed structure of aspera chaetominine A (**12**) and monocyclization product **26** in 31% and 45% yield, respectively. The spectral (^1^H and ^13^C NMR) data of our synthetic compound are different from those reported for the natural aspera chaetominine A, suggesting that the originally proposed stereochemistry for aspera chaetominine A (**12**) was incorrect. It is worth noting that compound **12** has been obtained in our previous investigation [65]. However, the ^1^H and ^13^C NMR spectra were recorded in DMSO-*d*_6_ which prevent a direct comparison with the data of aspera chaetominine A.

**Scheme 5:**
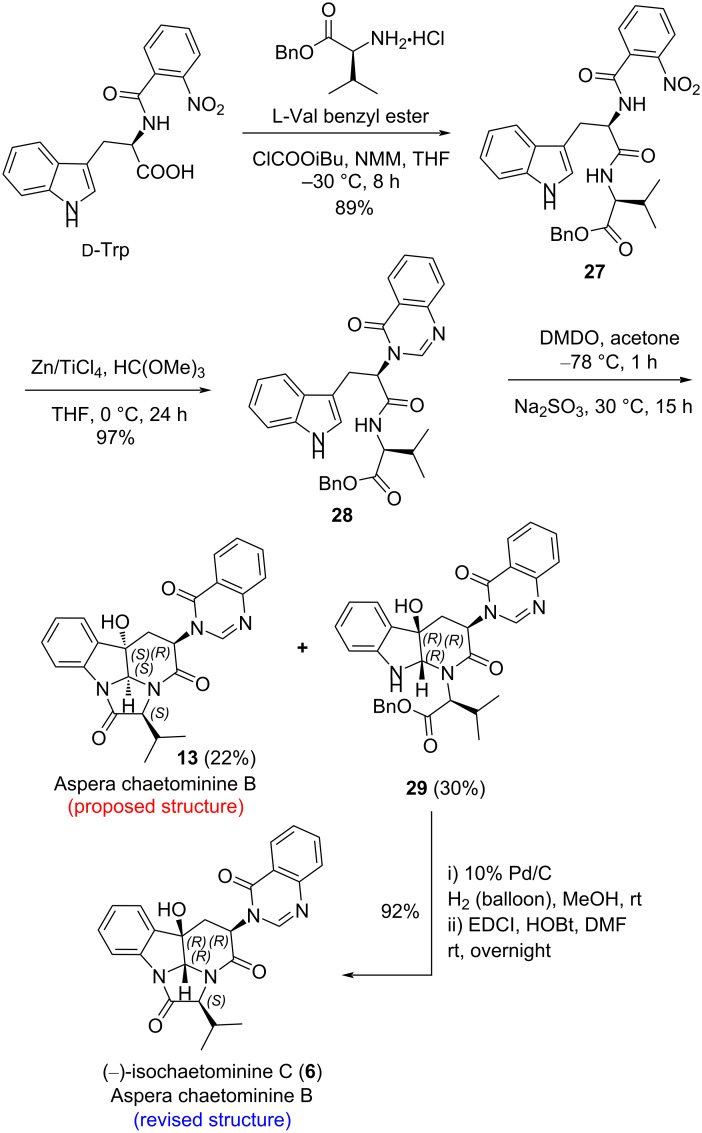
Enantioselective syntheses of both the proposed and revised structures of aspera chaetominine B.

We next addressed the synthesis of aspera chaetominine B (**13**). Employing our third-generation strategy featuring the employment of benzyl ʟ-valinate as the coupling component [63], tripeptide derivative **28** was synthesized in three steps. Exposure of **28** to DMDO in acetone followed by treating the resulting intermediates with Na_2_SO_3_ produced the proposed structure of aspera chaetominine B (**13**) and monocyclization product **29** in 22% and 30% yield, respectively (Scheme 5). Once again, a comparison of the ^1^H and ^13^C NMR data of our synthetic compound **13** with those of the natural aspera chaetominine B showed that two compounds are different, indicating a misassignment of the structure (**13**) for aspera chaetominine B. To our delight, one-pot catalytic debenzylation–lactamization of **29** afforded lactamization product (–)-isochaetominine C (**6**). The ^1^H and ^13^C NMR data of compound **6** matched those of natural aspera chaetominine B suggesting that aspera chaetominine B is (–)-isochaetominine C (**6**), whose absolute configuration is tentatively assigned as 2*R*,3*R*,11*S*,14*R*. It is worth noting that compound **13** has been obtained in our previous investigation [63]. However, the ^1^H and ^13^C NMR spectra were recorded in DMSO-*d*_6_ which prevent a direct comparison with the data of aspera chaetominine B.

## Conclusion

In summary, we have elaborated our epoxidation-triggered diastereodivergent approach to chaetominine-type alkaloids. As shown by the synthesis of (–)-isochaetominine A, the previously six-step total synthesis of 3,14-*cis* diastereomeric isochaetominines could be completed in five steps with higher yields. On the other hand, we have demonstrated that by simply alternating the quenching conditions for the key DMDO-epoxidation triggered double cyclization, one can realize: 1) a diastereoconvergent synthesis of two diastereomers of versiquinazoline H; 2) the enantiodivergent syntheses of racemic 14-*epi*-isochaetominine C, as well as (–)- and (+)-isochaetominine, respectively. This last-step enantiodivergent reaction allowed the one-step access to a racemic sample from a synthetic intermediate for determining the ee of a chaetominine-family alkaloid. Additionally, we have achieved the four-step enantioselective total synthesis of the proposed structure of aspera chaetominine A, and five-step enantioselective total synthesis of both the proposed and revised structures of aspera chaetominine B. The structure of aspera chaetominine B is revised to the known alkaloid (–)-isochaetominine C. Further application of this strategy to the total synthesis of natural products including the revised structure of aspera chaetominine A is in progress in our laboratory, and the results will be reported elsewhere in due course.

## Supporting Information

File 1 General methods and materials, experimental procedures, characterization data, and copies of ^1^H and ^13^C NMR spectra of compounds **19/20**, **25**, **26**, **12**, **13**, and **6**.

## Data Availability

All data that supports the findings of this study is available in the published article and/or the supporting information of this article. Crystallographic data for **19**/**20** has been deposited at the Cambridge Crystallographic Data Centre (CCDC 1905613) and can be obtained from https://www.ccdc.cam.ac.uk/structures/.
