# Correction: Further elaboration of the stereodivergent approach to chaetominine-type alkaloids: synthesis of the reported structures of aspera chaetominines A and B and revised structure of aspera chaetominine B

**DOI:** 10.3762/bjoc.22.119

**Published:** 2026-10-28

**Authors:** Jin-Fang Lü, Jiang-Feng Wu, Jian-Liang Ye, Pei-Qiang Huang

**Affiliations:** 1 Department of Chemistry, Fujian Provincial Key Laboratory of Chemical Biology, College of Chemistry and Chemical Engineering, Xiamen University, Xiamen, Fujian 361005, P. R. China

**Keywords:** epoxidation, selective epimerization, stereodivergent synthesis, structural revision, tandem reaction

The authors noticed an error in Scheme 5 of the original article: the text below the structure of the first molecule (top left corner), “ᴅ-Trp” should have read as “one-step from ᴅ-Trp”.

The corrected Scheme 5 of the original article is shown below in Scheme 1.

The authors apologize for any inconvenience caused by the originally published error in Scheme 5.

**Scheme 1:**
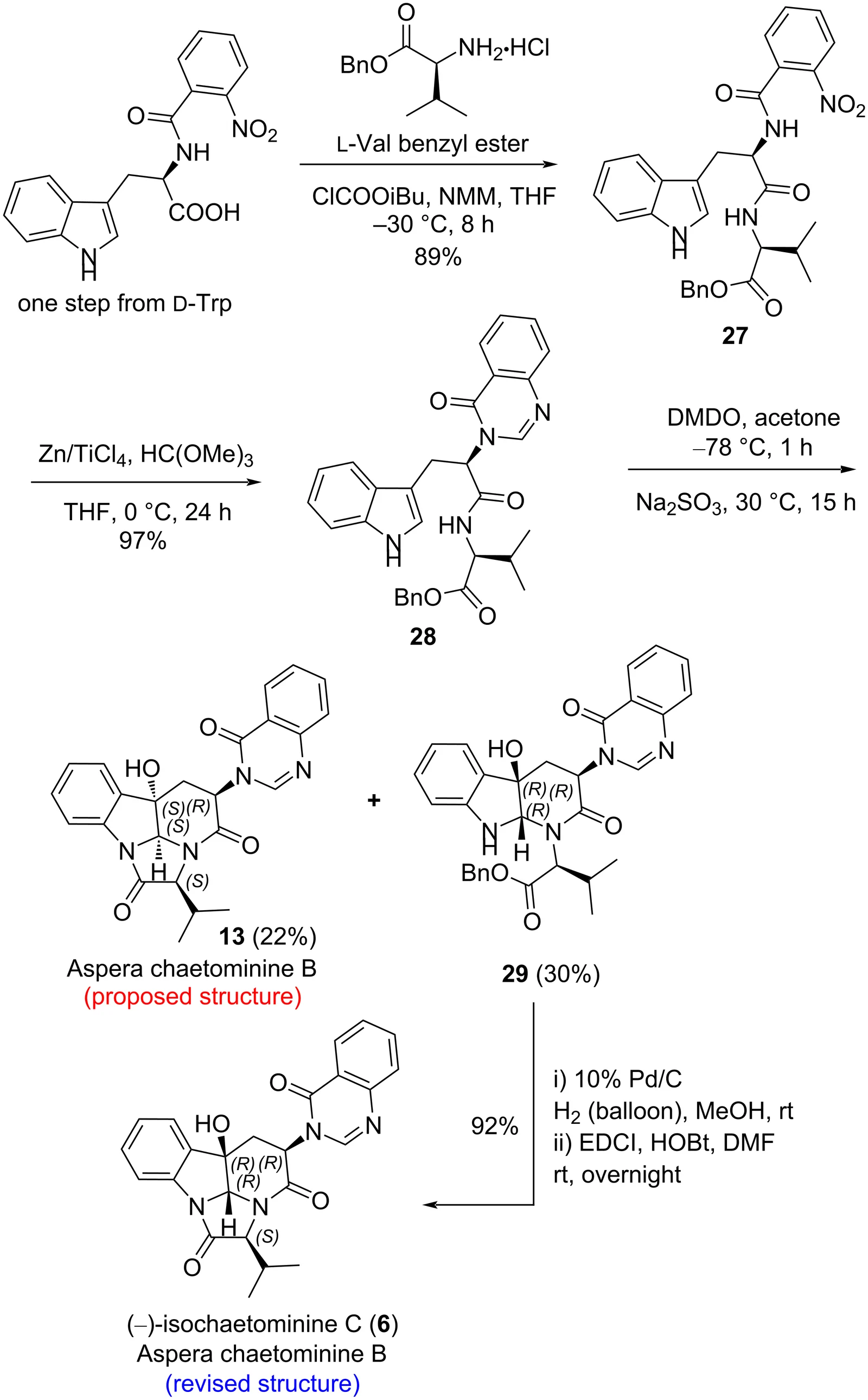
Corrected Scheme 5 of the original article. Enantioselective syntheses of both the proposed and revised structures of aspera chaetominine B.

