# Supplementary material for: Further elaboration of the stereodivergent approach to chaetominine-type alkaloids: synthesis of the reported structures of aspera chaetominines A and B and revised structure of aspera chaetominine B
Source: Beilstein J Org Chem. 2025 Oct 13;21:2072–81. doi: 10.3762/bjoc.21.162 (PMC12536460; doi:10.3762/bjoc.21.162)

BEILSTEIN JOURNAL OF ORGANIC CHEMISTRY

Supporting Information

for

Further elaboration of the stereodivergent approach to chaetominine-type alkaloids: synthesis of the reported structures of aspera chaetominines A and B and revised structure of aspera chaetominine B

Jin-Fang Lü, Jiang-Feng Wu, Jian-Liang Ye and Pei-Qiang Huang

Beilstein J. Org. Chem. **2025,** *21,* 2072–2081. doi:10.3762/bjoc.21.162

General methods and materials, experimental procedures, characterization data, and copies of ^{1}H and ^{13}C NMR spectra of compounds 19/20, 25, 26, 12, 13, and 6

License and Terms: This is a supporting information file under the terms of the Creative Commons Attribution License (https://creativecommons.org/licenses/by/4.0). Please note that the reuse, redistribution and reproduction in particular requires that the author(s) and source are credited and that individual graphics may be subject to special legal provisions.
The license is subject to the *Beilstein Journal of Organic Chemistry* terms and conditions: (https://www.beilstein-journals.org/bjoc/terms)

Table of contents

1. Experimental section S1
2. X-Ray structure of compound **19/20** S10
3. Chiral HPLC diagrams of compounds **(±)-7** and **(+)-7** S14
4. The comparison of ^{1}H NMR and ^{13}C NMR data of our synthetic (–)-isochaetominine C (**6**) with aspera chaetominine B S16
5. References S18
6. Copies of NMR spectra of **19/20, 25, 26, 12, 13, 6** S19

EXPERIMENTAL SECTION

General

Melting points were determined by a Switzerland Büchi M-560 automatic melting point apparatus. Infrared spectra were measured with a Nicolet Avatar 330 FT-IR spectrometer using film KBr pellet techniques. ^{1}H NMR and ^{13}C NMR spectra were recorded on 500 MHz spectrometer with $CDCl_3$ or CD_3OD as solvent. Chemical shifts (δ) are reported in ppm and respectively referenced to either the internal standard Me_4Si (TMS) or solvent signals (Me_4Si at 0 ppm for ^{1}H NMR, and $CDCl_3$ at 77.0 ppm, DMSO-d_6 at 40.0 ppm or CD_3OD at 49.0 ppm for ^{13}C NMR). HRMS spectra were recorded on a Bruker Dalton Esquire 3000 plus mass spectrometer by the ESI method. Chiral HPLC analysis was performed on an Agilent LC1260 instrument. Unless otherwise stated, reactions were performed in oven-dried glassware under a nitrogen atmosphere using standard Schlenk techniques. Flash column chromatography was performed with silica gel (300–400 mesh), eluting with EtOAc/petroleum ether. THF used in the reactions was dried by distillation over metallic sodium and benzophenone; dichloromethane was distilled from calcium hydride; anhydrous acetone employed was dried using 4 Å molecular sieves. All other commercially available compounds were used as received.

General procedure for step 1

The procedures for the preparation of (2-nitrobenzoyl)-D-tryptophan from D-tryptophan by *N*-aroylation with **23** have been described in our previous reports [1,2].

General procedure for step 2 (general procedure 2)

To a solution of (2-nitrobenzoyl)-D-tryptophan (1.02 mmol) in THF (4 mL) at −30 °C NMM (0.17 mL, 1.53 mmol) and iBuOCOC1 (0.15 mL, 1.12 mmol) were added successively. After being stirred at −30 °C under Ar atmosphere for 1 h, a solution of an amino acid methyl ester hydrochloride acid salt (2.04 mmol) and NMM (0.34 mL, 3.06 mmol) in THF (7 mL) was added slowly to the resulting suspension. The mixture was stirred for 8 h, then quenching with a saturated aqueous solution of NH_4Cl (5 mL).

The resulting mixture was diluted with water (20 mL) and the phases separated. The aqueous phase was extracted with EtOAc (10 mL × 3). The combined organic phases were dried over anhydrous Na_2SO_4, filtered and concentrated under reduced pressure. The residue was purified by flash chromatography on silica gel to give the dipeptide derivative.

Benzyl (*S*)-2-((*R*)-3-(1*H*-indol-3-yl)-2-(2-nitrobenzamido)propanamido)-3-methylbutanoate (27).

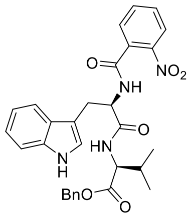

27

Following the general procedure 2, the reaction of compound (2-nitrobenzoyl)-D-tryptophan (99 mg, 0.28 mmol) gave, after flash chromatography on silica gel (eluent: EtOAc: PE = 1:1), compound **27** (135 mg, yield: 89%) as white solid. The ^{1}H NMR, ^{13}C NMR data are consistent with those reported in the literature [1].

General procedure for step 3 (general procedure 3)

To a mixture of zinc powder (517 mg, 7.96 mmol) and THF (33 mL), $TiCl_4$ (0.44 mL, 3.99 mmol) was added at 0 ºC. The resulting mixture was heated to 50 °C and stirred for 1 h. After cooling to 0 °C, a solution of the tryptophan-derived dipeptide (1.00 mmol) in THF (6.7 mL) and trimethylorthoformate (0.44 mL, 3.99 mmol) were added successively. The mixture was stirred at 0 °C for 24 h. Brine (6 mL) was then added to the reaction mixture, and the resulting suspension was stirred for 2 h. Following phase separation, the aqueous phase was extracted with EtOAc (20 mL × 3). The combined organic phases were dried over anhydrous Na_2SO_4, filtered, and concentrated under reduced pressure. The residue was purified by flash chromatography on silica gel to give the corresponding quinazolino-dipeptide derivative.

Methyl ((*R*)-3-(1*H*-indol-3-yl)-2-(4-oxoquinazolin-3(4*H*)-yl)propanoyl)-L-isoleucinate (25)

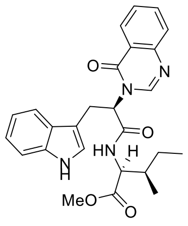

25

Following the general procedure 1-3, the reaction of compound D-Trp (61 mg, 0.3 mmol), **23** and **24** gave, after flash chromatography on silica gel (eluent: EtOAc:PE = 1:1), compound **25** (110 mg, yield: 80%) as white solid.

25: Mp 84-86 °C (eluent : EtOAc, hexane); $[\alpha]_D^{20}$ 52.38 (*c* 1.0, $CHCl_3$); ^{1}H NMR (500 MHz, $CDCl_3$) δ 8.44 (s, 1H), 8.30 (s, 1H), 8.24 (d, *J* = 8.1 Hz, 1H), 7.74 – 7.64 (m, 3H), 7.43 (ddd, *J* = 8.2, 6.8, 1.6 Hz, 1H), 7.30 (d, *J* = 8.1 Hz, 1H), 7.16 (ddd, *J* = 8.1, 7.0, 1.2 Hz, 1H), 7.09 (ddd, *J* = 8.0, 7.1, 1.0 Hz, 1H), 7.03 (d, *J* = 2.4 Hz, 1H), 6.89 (d, *J* = 8.5 Hz, 1H), 5.94 (dd, *J* = 8.9, 7.0 Hz, 1H), 4.45 (dd, *J* = 8.4, 5.0 Hz, 1H), 3.74 (dd, *J* = 14.5, 9.0 Hz, 1H), 3.55 (s, 3H), 3.44 (dd, *J* = 14.5, 7.0 Hz, 1H), 1.72 – 1.63 (m, 1H), 1.27 – 1.14 (m, 1H), 0.99 – 0.86 (m, 1H), 0.78 (t, *J* = 7.3 Hz, 3H), 0.66 (d, *J* = 6.9 Hz, 3H). ^{13}C NMR (125 MHz, $CDCl_3$) δ 171.5, 168.7, 161.3, 147.5, 144.3, 136.3, 134.5, 127.5, 127.2, 126.9, 126.9, 123.3, 122.4, 121.5, 119.9, 118.5, 111.3, 109.5, 57.0, 56.4, 52.1, 37.5, 27.5, 25.0,15.1, 11.5. IR (neat, cm^{-1}): 3318, 2924, 1742, 1658, 1260; HRMS (ESI, *m/z*) calcd for $C_{26}H_{28}N_4O_4Na$ (M+Na^+): 483.2003, found: 483.2015.

Benzyl ((*R*)-3-(1*H*-indol-3-yl)-2-(4-oxoquinazolin-3(4*H*)-yl)propanoyl)-L-valinate (28)

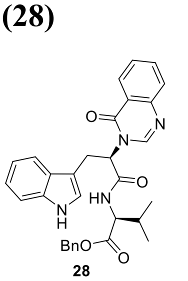

28

Following the general procedure 3, the reaction of compound **27** (163 mg, 0.3 mmol) gave, after flash chromatography on silica gel (eluent: EtOAc: PE = 1:1), compound **28**

(152 mg, yield: 97%) as white solid. The ^{1}H NMR, ^{13}C NMR data are consistent with those reported in the literature [1].

General procedure for step 4 (general procedure 4-A)

In a manner similar to our previous work [1], to a solution of the quinazolinonyl dipeptide derivative (0.28 mmol) in anhydrous acetone (2 mL), DMDO (0.04 M in acetone, 14 mL, 0.56 mmol) was added at −78 °C. After being stirred for 1 h, saturated aqueous solution of Na_2SO_3 (10 mL) was added and the resulting mixture was stirred at 0 °C for 10 min, then warmed to 45 ºC and stirred for 2 h. The reaction was quenched with a saturated aqueous solution of NH_4Cl (10 mL) and concentrated under reduced pressure. To the resulting residue was added H_2O (50 mL), and the mixture was extracted with EtOAc (30 mL × 3). The combined organic layers were washed with brine (2 mL), dried over anhydrous Na_2SO_4, filtered, and concentrated under reduced pressure. The residue was purified by flash chromatography on silica gel to give an isochaetominine-type compound, or versiquinazoline H-type compound and a monocyclization product.

Aspera chaetominine A (proposed structure, 12) and 26

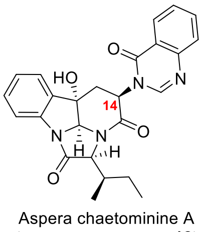

Aspera chaetominine A
(Proposed structure, **12**)

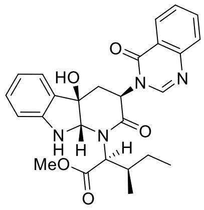

26

Following general procedure 4-A, the reaction of compound **25** (129 mg, 0.28 mmol) gave, after flash chromatography on silica gel (eluent: EtOAc:PE = 1:1 to 2:1), compound **12** (38 mg, yield: 31%) as white crystal and **26** (60 mg, yield: 45%) as white solid. The ^{1}H NMR, ^{13}C NMR data used DMSO-d_6 are consistent with those reported in the literature [2].

12: ^{1}H NMR (500 MHz, Methanol-d_4) δ 8.21 (*app.* d, J = 6.4 Hz, 2H), 7.82 (ddt, J = 8.5, 4.7, 1.5 Hz, 1H), 7.68 (dd, J = 8.2, 2.4 Hz, 1H), 7.55 (d, J = 8.1 Hz, 2H), 7.46 (d,

J = 7.6 Hz, 1H), 7.40 (t, J = 7.8 Hz, 1H), 7.22 (t, J = 7.6 Hz, 1H), 5.49 (s, 1H), 4.58 (d, J = 3.0 Hz, 1H), 3.10 – 2.85 (m + *br.* s , 2H), 2.66 (d, J = 12.7 Hz, 1H), 1.69 (*app.* quintet, J = 7.5 Hz, 2H), 0.94 (td, J = 7.5, 1.7 Hz, 3H), 0.83 (d, J = 6.8 Hz, 3H) (resonance of H14 was not observed due to slow hindered rotation of C14-N bond) [2]; ^{13}C NMR (125 MHz, Methanol-d_4) δ 172.8, 167.6, 162.4, 148.7, 148.3, 140.8, 136.8, 136.0, 131.4, 128.7, 128.0, 127.7, 126.8, 125.8, 123.1, 115.9, 84.3, 78.2, 68.4, 61.1 (broad weak resonance of C14 due to slow hindered rotation of C14-N bond) [2], 39.5, 34.0, 26.3, 14.2, 12.9.

26: Mp 125-127 °C (EtOAc, hexane); $[\alpha]_D^{20}$ 194.3 (*c* 1.0, $CHCl_3$); ^{1}H NMR (500 MHz, $CDCl_3$) δ 8.19 (dd, J = 8.0, 1.5 Hz, 1H), 7.71 (ddd, J = 8.5, 7.0, 1.6 Hz, 1H), 7.63 (d, J = 7.7 Hz, 1H), 7.50 (br s, 1H), 7.44 (t, J = 7.6 Hz, 1H), 7.29 (dd, J = 7.5Hz, 1H), 7.16 (t, J = 7.5Hz,1H), 6.79 (t, J = 7.5 Hz, 1H), 6.67 (d, J = 7.9 Hz, 1H), 5.49 (d, J = 4.5Hz, 1H), 5.17 (d, J = 4.5 Hz, 1H), 5.03 (d, J = 10.6 Hz, 1H), 3.88 (br s, 1H), 3.76 (s, 3H), 2.89 (br s, 1H), 2.47 (dd, J = 12.1, 3.9 Hz, 1H), 2.10 – 1.98 (m, 1H), 1.82 (s, 1H), 1.77 – 1.67 (m, 1H), 1.45 – 1.32 (m, 1H), 1.03 – 0.90 (m, 6H).^{13}C NMR (125 MHz, $CDCl_3$) δ 173.8, 170.5, 160.5, 148.4, 147.2, 145.2, 134.4, 130.9, 129.2, 127.3, 127.1, 127.0, 123.8, 121.8, 120.3, 110.3, 80.1, 79.8, 59.0, 52.2, 41.1, 34.6, 24.6, 15.4, 10.3 (resonance of C14 was not observed due to slow hindered rotation of C14-N bond) [2]. IR (neat, cm^{-1}): 3389, 2924, 1683, 1612, 1471, 1228, 1174; HRMS (ESI, *m/z*) calcd for $C_{26}H_{28}N_4O_5Na$ (M+Na$^+$): 499.1952, found: 499.1945.

Aspera chaetominine B (Proposed structure, 13) and 29

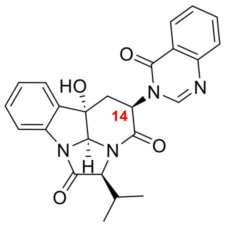

Aspera chaetominine B
(Proposed structure, **13**)

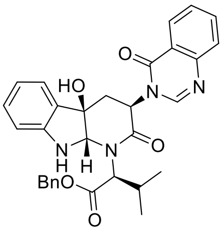

29

Following general procedure 4-A, the reaction of compound **28** (146 mg, 0.28 mmol) gave, after flash chromatography on silica gel (eluent: EtOAc:PE = 1:1 to 2:1),

compound **13** (26 mg, yield: 22%) as white solid and **29** (45 mg, yield: 30%) as white solid. The ^{1}H NMR, ^{13}C NMR data used DMSO-d_6 are consistent with those reported in the literature [1].

13: ^{1}H NMR (500 MHz, Methanol-d_4) δ 8.27 – 8.15 (m, 2H), 7.87 – 7.78 (m, 1H), 7.69 (dd, J = 7.9, 4.8 Hz, 1H), 7.56 (t, J = 7.7 Hz, 2H), 7.46 (d, J = 7.6 Hz, 1H), 7.41 (tdd, J = 7.8, 3.1, 1.5 Hz, 1H), 7.26 – 7.17 (m, 1H), 5.49 (s, 1H), 4.49 (s, 1H), 3.31 (m, 1H), 2.80 – 3.20 (*br.* s, 1H), 2.65 (d, J = 12.7 Hz, 1H), 1.21 (d, J = 7.2 Hz, 1H), 0.87 (d, J = 6.8 Hz, 1H), (resonance of H14 was not observed due to slow hindered rotation of C14-N bond) [2]; ^{13}C NMR (125 MHz, Methanol-d_4) δ 172.9, 167.7, 162.4, 148.7, 148.6, 140.9, 136.9, 136.0, 131.4, 128.8, 128.0, 127.7, 126.8, 125.8, 123.4, 115.9, 84.3, 78.3, 69.4, 60.2 (broad weak resonance of C14 due to slow hindered rotation of C14-N bond) [2], 39.5, 27.2, 18.4, 16.5.

General procedure for step 4 (general procedure 4-B)

To a solution of a quinazolinonyl dipeptide derivative (0.28 mmol) in anhydrous acetone (2 mL), DMDO (0.04 M in acetone, 14 mL, 0.56 mmol) was added at −78 °C. After being stirred for 1 h, K_2CO_3/MeOH (94 mg/10 mL, stood at rt overnight, pH = 11) was added to the mixture, which was subsequently warmed to 35 °C over a period of 30 min. The reaction was quenched with a saturated aqueous solution of NH_4Cl (10 mL) and concentrated under reduced pressure. Water (50 mL) was added to the resulting residue, and the mixture was extracted with EtOAc (30 mL × 3). The combined organic layers were washed with brine (2 mL), dried over anhydrous Na_2SO_4, filtered, and concentrated under reduced pressure. The residue was purified by flash chromatography on silica gel to give two kinds of versiquinazoline H-type compounds.

(2*S*,3*S*,11*R*,14*R*,28*S*)-2,3,11-tris-*epi*-versiquinazoline H (19) and (2*R*,3*R*,11*S*,14*S*,28*S*)-14-*epi*-versiquinazoline H (20)

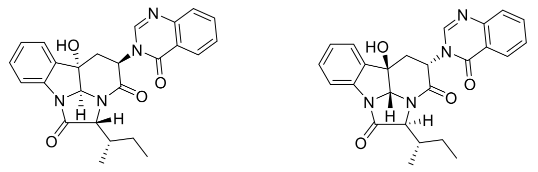

19 20

Following general procedure 4-B, the reaction of compound **17** (150 mg, 0.28 mmol) gave, after flash chromatography on silica gel (eluent: EtOAc:PE = 1:2 to 1:1), **19** and **20** (white crystals, 93 mg, yield: 75%) as inseparable compounds. The reaction of compound **18** (150 mg, 0.28 mmol) also yielded **19** and **20** (90 mg, yield: 73%).

19 and **20**: Mp 270-272 °C (EtOAc, hexane); $[\alpha]_D^{20}$ −7.8 (*c* 0.1, CH_3OH); ^1H NMR (500 MHz, DMSO-d_6) δ 8.24 (d, *J* = 5.4 Hz, 1H), 8.21 (d, *J* = 8.0 Hz, 1H), 7.87 (ddd, *J* = 8.4, 7.2, 1.6 Hz, 1H), 7.71 (d, *J* = 8.1 Hz, 1H), 7.59 (t, *J* = 7.6 Hz, 1H), 7.50 (dd, *J* = 7.7, 3.8 Hz, 1H), 7.48 (d, *J* = 7.7 Hz, 1H), 7.42 (t, *J* = 7.7 Hz, 1H), 7.25 (t, *J* = 7.5 Hz, 1H), 6.74 (s, 1H), 6.00 (br s, 1H), 5.79 (d, *J* = 10.2 Hz, 1H), 4.54 (d, *J* = 5.5 Hz, 0.5H), 4.45 (d, *J* = 7.1 Hz, 0.5H), 2.95 (*app*. t, *J* = 13.1 Hz, 1H), 2.49 – 2.47 (m, 1H), 2.06 – 1.96 (m, 1H), 1.70 – 1.58 (m, 1H), 1.36 – 1.23 (m, 1H), 1.12 – 1.01 (m, 3H), 0.98 – 0.90 (m, 3H). Integration of the resonances at 4.54 and 4.45 allowed determining the diastereomeric ratio of **19**/ **20** as 1:1. ^{13}C NMR (125 MHz, DMSO-d_6) δ 170.5, 170.1, 168.0, 167.6, 160.4, 147.8, 147.2, 138.1, 138.04, 137.98, 135.2, 130.1, 127.69, 127.67, 126.9, 126.0, 125.03, 125.02, 121.5, 115.23, 115.17, 85.4, 84.8, 77.23, 77.22, 69.2, 69.0, 68.8, 38.7, 38.6, 37.5, 37.0, 26.3, 26.0, 15.9, 15.8, 11.8, 11.7. IR (neat, cm^{-1}): 3428, 2922, 1713, 1681, 1612, 1481, 1325, 1293, 1132, 1076; HRMS (ESI, *m/z*) calcd for $C_{25}H_{24}N_4O_4Na$ (M+Na$^+$): 467.1690, found: 467.1696.

(+)-(2*R*,3*R*,11*S*,14*S*)-11-*epi*-Isochaetominine C ((+)-7) and (−)-(2*S*,3*S*,11*R*,14*R*)-3,4,14-tris-*epi*-isochaetominine C ((−)-7)

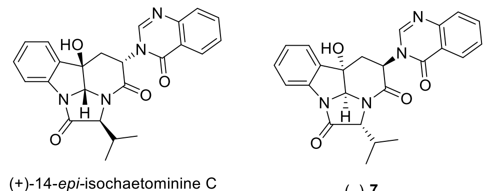

(+)-14-*epi*-isochaetominine C
(+)-**7**

(−)-**7**

Following general procedure 4-B, the reaction of compound **21** (146 mg, 0.28 mmol) gave, after flash chromatography on silica gel (eluent: EtOAc:PE = 1:2 to 1:1), (+)-**7**

and (−)-**7** (white solid, 90 mg, yield: 75%) as a racemate. The ^{1}H NMR, ^{13}C NMR data are consistent with those reported in the literature [1].

(−)-(2*S*,3*S*,11*R*,14*R*)-Isochaetominine [(−)-10]

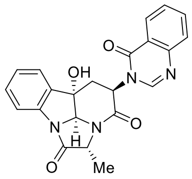

(–)-isochaetominine ((–)-**10**)

To a solution of a compound **22** (0.1 mmol) in anhydrous acetone (2 mL), K_2CO_3/MeOH (19 mg/2 mL, stood at rt overnight, pH = 11) was added and stirred at 35 ºC for 30 min. The reaction was quenched with a saturated aqueous solution of NH_4Cl (5 mL) and concentrated under reduced pressure. Water (10 mL) was added to the resulting residue, and the mixture was extracted with EtOAc (10 mL × 3). The combined organic layers were washed with brine (2 mL), dried over anhydrous Na_2SO_4, filtered, and concentrated under reduced pressure. The residue was purified by flash chromatography on silica gel (eluent: EtOAc:PE = 3:2) to give compound (−)-**10** (28 mg, 71%) as white solid. The ^{1}H NMR, ^{13}C NMR data are consistent with those reported in the literature [1].

General procedure for step 5

In a manner similar to our previous work [2], a suspension of a quinazolinonyl dipeptide derivative (0.10 mmol) and 10% Pd/C (8 mg) in methanol (2 mL) was stirred under an atmosphere of H_2 for 2 h at room temperature. The reaction mixture was filtered through a celite pad and the residue was washed with methanol. The filtrate was concentrated under reduced pressure to get the carboxylic acid. Without further purification, the residue was treated directly with HOBt (40 mg, 0.3 mmol) and EDCI (115 mg, 0.6 mmol) in anhydrous CH_2Cl_2 (2 mL) and DMF (0.1 mL) at room temperature and stirred for 12 h. The reaction was quenched with water (5 mL), and the resulting mixture was extracted with CH_2Cl_2 (10 mL × 3). The combined organic layers were washed with brine (2 mL), dried over anhydrous Na_2SO_4, filtered, and concentrated under reduced

pressure. The residue was purified by flash chromatography on silica gel to give the corresponding isochaetominine-type compound or versiquinazoline H-type compound.

(−)-(2*R*,3*R*,11*S*,14*R*)-Isochaetominine A (4)

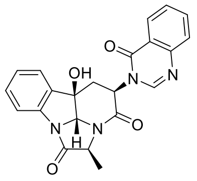

(−)-isochaetominine A (**4**)

Following general procedure 5, the reaction of compound **14** (51 mg, 0.10 mmol) gave, after flash chromatography on silica gel (eluent: EtOAc:PE = 1:1 to 2:1), compound **4** (37 mg, yield: 91%) as a white solid. The ^{1}H NMR, ^{13}C NMR data are consistent with those reported in the literature [1].

(−)-(2*S*,3*S*,11*S*,14*S*)-3,4,14-tris-*epi* -Isochaetominine C (16)

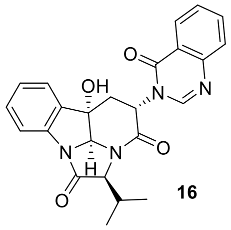

16

Following general procedure 5, the reaction of compound **15** (53 mg, 0.10 mmol) gave, after flash chromatography on silica gel (eluent: EtOAc:PE = 1:2 to 2:1), compound **16** (40 mg, yield: 92%) as a white solid. The ^{1}H NMR, ^{13}C NMR data are consistent with those reported in the literature [1].

(−)-(2*R*,3*R*,11*S*,14*R*) -Isochaetominine C (6)

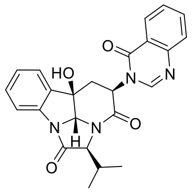

(−)-isochaetominine C (**6**)

Following general procedure 5, the reaction of compound **29** (53 mg, 0.10 mmol) gave, after flash chromatography on Silica gel (eluent: EtOAc:PE = 1:2 to 2:1), compound **6**

(39 mg, yield: 92%) as a white solid. The ^{1}H NMR, ^{13}C NMR data are consistent with those reported in the literature [2].

6: ^{1}H NMR (500 MHz, Methanol-d_4) δ 8.27 (dd, J = 8.2, 1.7 Hz, 1H), 8.25 (s, 1H), 7.87 (m, 1H), 7.72 (d, J = 8.1 Hz, 1H), 7.61 (d, J = 7.8 Hz, 1H), 7.57 (d, J = 7.3 Hz, 1H), 7.54 (s, 1H), 7.44 (td, J = 7.6, 1.4 Hz, 1H), 7.28 (td, J = 7.4, 1.2 Hz, 1H), 5.89 (s, 1H), 4.93 (dd, J = 7.7, 5.2 Hz, 1H), 4.40 (d, J = 8.9 Hz, 1H), 3.15 (dd, J = 14.3, 7.7 Hz, 1H), 2.72 (dd, J = 14.3, 5.2 Hz, 1H), 2.57 – 2.42 (m, 1H), 1.27 (t, J = 6.8 Hz, 6H); ^{13}C NMR (125 MHz, Methanol-d_4) δ 175.4, 167.1, 162.1, 148.8, 147.9, 141.8, 136.0, 135.8, 131.7, 128.8, 128.0, 127.6, 126.8, 125.4, 123.2, 116.1, 85.9, 75.6, 71.5, 58.0, 35.8, 30.4, 20.7, 19.4.

Crystal structure determination of compound 19/ 20.

CCDC 1905613 contains the crystallographic data for compound **19/ 20**[3]. These data can be obtained free of charge from The Cambridge Crystallographic Data Centre via www.ccdc.cam.ac.uk/structures**.**

Structure determination Single crystals of **19/ 20** were obtained by slow evaporation of a mixed solution in MeOH, CH_2Cl_2 and *n*-hexane. A suitable crystal was selected and measured with Mo$K\alpha$ radiation (λ = 0.71073 Å) on a Bruker SMART APEX-CCD diffractometer using a ψ-ω scan mode. The crystal was kept at 273.15 K during data collection. A total of 19407 reflections were collected in the range of 3.12° < 2θ <50°, and 7625 were independent (R_{int} = 0.0366, R_{sigma} = 0.0612). Lattice determination and data collection were carried out using Bruker SMART software. Date reduction was performed with SAINT version 6.28A. Using Olex2[3], the structure was solved with the olex2.solve[4] structure solution program using Charge Flipping and refined with the olex2.refine[4] refinement package using Gauss-Newton minimisation. The non-hydrogen atoms were refined anisotropically, and hydrogen atoms were determined with theoretical calculation.

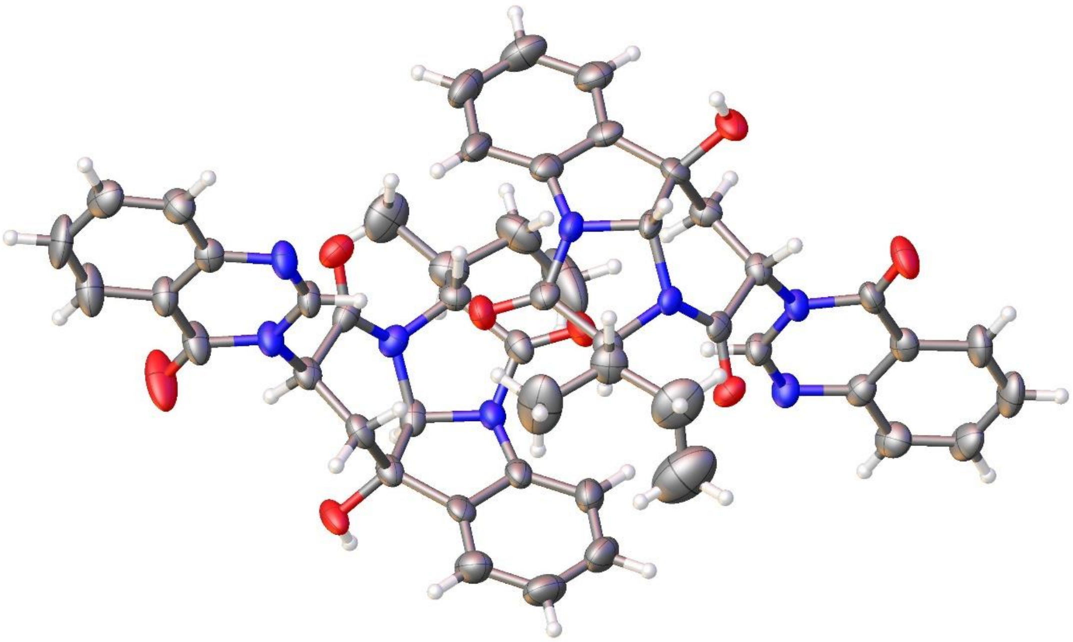

Figure S1 The thermal ellipsoid plot of **19**/ **20** at 50% probability

Table S1. Crystal data and structure refinement for 19/ 20.

Identification code	h0523m
Empirical formula	C25 H24 N4 O4
Formula weight	444.48
Temperature/K	273.15
Crystal system	monoclinic
Space group	$P2_1$
a/Å	10.5361(16)
b/Å	15.952(3)
c/Å	13.882(2)
α/°	90
β/°	109.384(3)
γ/°	90
Volume/Å^3	2200.8(6)
Z	44
ρ_{calc}g/cm^3	1.3414
μ/mm^{-1}	0.093
F(000)	936.5
Crystal size/mm^3	0.4 × 0.3 × 0.2
Radiation	Mo Kα (λ = 0.71073)
2Θ range for data collection/°	3.12 to 50
Index ranges	$-14 \le h \le 14$, $-20 \le k \le 20$, $-18 \le l \le 18$

Reflections collected	19407
Independent reflections	7625 [R_{int} = 0.0366, R_{sigma} = 0.0612]
Data/restraints/parameters	7625/1/601
Goodness-of-fit on F^2	1.057
Final R indexes [I>=2σ (I)]	R_1 = 0.0630, wR_2 = 0.1432
Final R indexes [all data]	R_1 = 0.0696, wR_2 = 0.1475
Largest diff. peak/hole / e $Å^{-3}$	0.61/-0.23
Flack parameter	1(3)

Table S2 Fractional Atomic Coordinates (×10^4) and Equivalent Isotropic Displacement Parameters (Å^2×10^3) for 19/20. $U_{eq}$is defined as 1/3 of of the trace of the orthogonalised U_{IJ}tensor.

Atom	*x*	*y*	*z*	U(eq)
O001	11242(3)	4523.3(18)	3555.2(19)	37.4(6)
O002	3592(3)	5397.3(19)	1581(2)	43.7(7)
O003	6486(3)	4500.7(18)	1529(2)	42.6(7)
O004	13015(3)	3401.4(19)	6453(2)	50.6(8)
O005	9001(3)	2394.2(18)	5013(2)	45.6(7)
N006	7220(3)	3487(2)	2743(2)	34.2(7)
O007	5828(3)	7556.5(17)	170(2)	45.4(7)
O008	8315(3)	5486.9(19)	3682(2)	46.2(7)
N009	11652(3)	4434.7(19)	5566(2)	31.2(7)
N00A	9469(3)	3646.1(18)	3112(2)	27.5(6)
N00B	5317(3)	6302(2)	2043(2)	34.1(7)
N00C	2742(3)	4061(2)	-739(2)	39.2(8)
N00D	3219(3)	5491(2)	-422(2)	38.0(8)
N00E	12224(3)	5869(2)	5829(2)	35.3(8)
C00F	12827(4)	4142(3)	6293(3)	34.7(9)
N00G	7574(3)	6497.5(19)	2453(2)	33.7(7)
C00H	7839(4)	6669(2)	891(3)	36.0(9)
C00I	7366(4)	4046(3)	2057(3)	34.7(9)
C00J	10519(4)	4034(2)	3803(3)	30.2(8)
C00K	8479(4)	3137(2)	4479(3)	34.6(9)
C00L	8808(4)	3995(2)	2089(3)	31.6(8)
C00M	1467(4)	4273(2)	-1381(3)	35.7(9)
C00N	4146(4)	6095(2)	243(3)	34.2(9)
O00O	1784(4)	6507(2)	-1254(3)	85.0(13)
C00P	526(4)	3650(3)	-1776(3)	44.5(10)
C00Q	3502(4)	4659(3)	-303(3)	36.4(9)

C00R	11408(4)	5276(2)	5399(3)	34.1(9)
C00S	6321(4)	3504(3)	3302(3)	39.5(9)
C00T	5962(4)	5949(3)	3073(3)	34.0(9)
C00U	6292(4)	6838(3)	1792(3)	36.9(9)
C00V	13464(4)	5629(3)	6514(3)	35.1(9)
C00W	8499(3)	3123(2)	3373(3)	29.8(8)
C00X	13780(4)	4797(3)	6779(3)	35.9(9)
C00Y	9351(4)	3850(2)	5083(3)	33.7(9)
C00Z	6341(4)	6815(2)	708(3)	34.6(9)
C010	10697(4)	3840(2)	4920(3)	31.5(8)
C011	15032(4)	4594(3)	7481(3)	45.9(11)
C012	-743(4)	3850(3)	-2376(3)	50.1(11)
C013	8493(4)	6468(3)	1910(3)	36.3(9)
C014	5499(4)	6109(2)	92(3)	36.4(9)
C015	7437(4)	5934(2)	3138(3)	35.1(9)
C016	4301(4)	5894(3)	1352(3)	35.3(9)
C017	4996(4)	3727(3)	2967(4)	46.0(11)
C018	1100(4)	5101(3)	-1597(3)	41.2(10)
C019	6349(5)	3304(3)	4994(4)	50.8(11)
C01A	15970(4)	5197(4)	7868(3)	55.3(13)
C01B	7011(4)	3298(3)	4306(3)	38.8(10)
C01C	4998(5)	3511(4)	4675(4)	65.2(15)
C01D	9842(4)	6287(3)	2277(3)	45.0(10)
C01E	8520(5)	6688(3)	231(3)	50.6(12)
C01F	5661(5)	6432(3)	3929(3)	49.6(11)
C01G	8975(5)	3470(3)	1218(3)	47.5(11)
C01H	2036(5)	5766(3)	-1109(3)	47.7(11)
C01I	4338(4)	3728(3)	3680(4)	59.4(13)
C01J	14432(4)	6246(3)	6918(3)	46.8(11)
C01K	10524(4)	6296(3)	1593(4)	54.2(12)
C01L	10433(6)	3313(4)	1334(4)	77.5(18)
C01M	-1096(4)	4670(4)	-2619(3)	59.7(14)
C01N	6510(6)	6118(3)	4977(3)	63.2(14)
C01O	15664(4)	6031(3)	7576(3)	50.9(12)
C01P	-194(5)	5303(3)	-2225(4)	60.5(14)
C01Q	9880(5)	6487(3)	568(4)	59.8(13)
C01R	4183(5)	6341(4)	3786(4)	71.2(15)
C01S	8170(6)	3853(5)	189(3)	89(2)
C01T	7692(8)	6661(4)	5480(5)	123(3)
C01U	11149(7)	4010(5)	1190(6)	123(3)

Chiral HPLC diagrams of compounds (±)-7 and (+)-7

The enantiomeric excess (*ee*) of compounds (+)-**7** and (–)-**7** was determined by HPLC analysis (column, Chiralpak AD-H 4.6 × 250 mm; *n*-hexane/ iPrOH= 50:50; flow rate: 1.0 mL/min) t_R (min): (+)-**7**: 13.712 (99.3%), (–)-**7**: 10.522 (0.6%), *ee* = 98.7%.

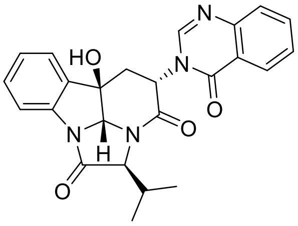

(+)-14-*epi*-isochaetominine C
(+)-**7**

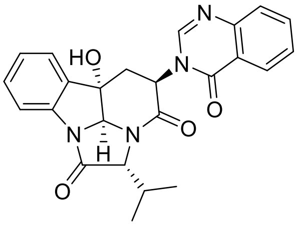

(–)-**7**

HPLC analysis of (±)-**7**

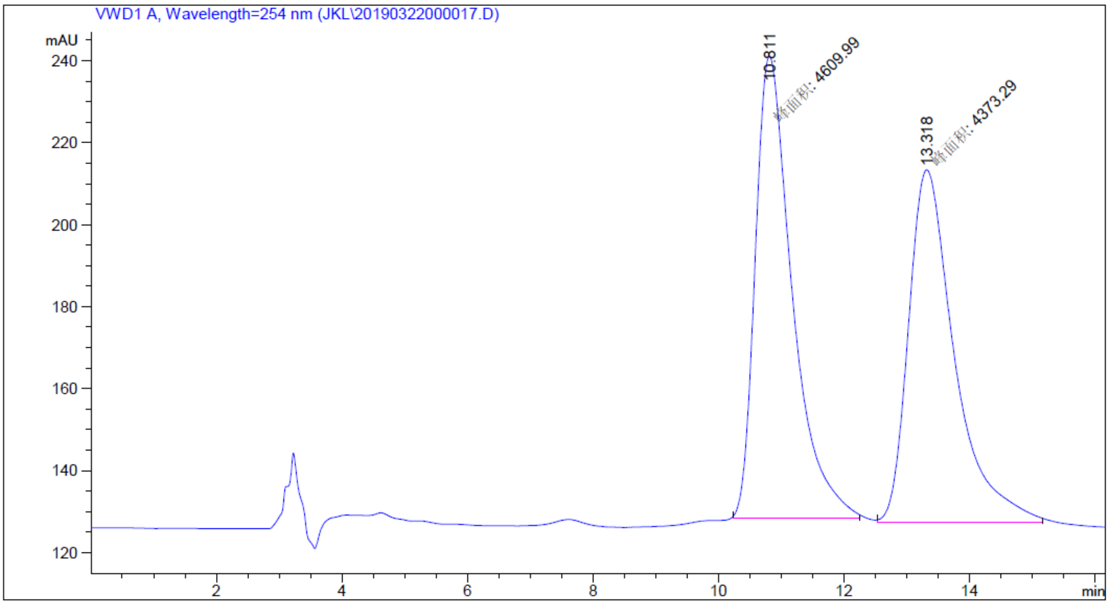

Peak #	RetTime [min]	Yype	Area [Mau*s]	Height [mAU]	Area %
1	10.811	MM	4609.99072	112.72957	51.3175
2	13.318	MM	4373.28760	86.02417	48.6825
Total			8983.27832	198.75374	

HPLC analysis of **(+)-7**

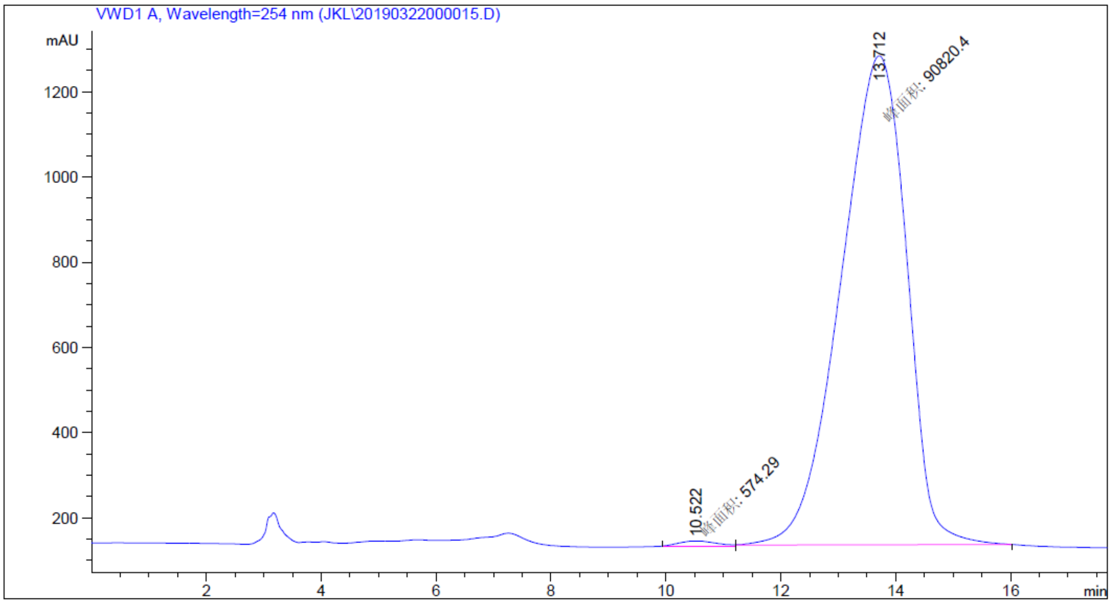

Peak #	RetTime [min]	Yype	Area [Mau*s]	Height [mAU]	Area %
1	10.522	MM	574.29022	12.51264	0.6284
2	13.712	MM	9.08204e4	1147.79028	99.3716
Total			9.13947e4	1160.30292	

The comparison of ^{1}H NMR and ^{13}C NMR data of our synthetic (–)-isochaetominine C (6) with Aspera chaetominine B.

Table S3. Comparison of ^{1}H NMR data of our synthetic (–)-isochaetominine C (6) with Aspera chaetominine B

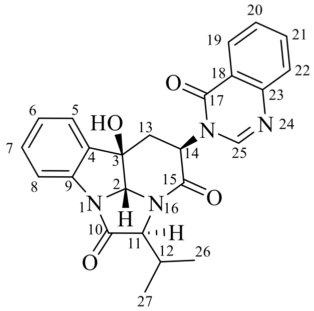

(–)-isochaetominine C (**6**)

Position	Aspera chaetominine B [3] ^{1}H NMR (500 MHz, Methanol-d_4)	This work ^{1}H NMR (500 MHz, Methanol-d_4)	Δ (ppm)
2	5.86 (1H, s)	5.89 (s, 1H)	-0.03
3-OH	-	-	-
5	7.60 (1H, d, J = 7.8)	7.57 (d, J = 7.3 Hz, 1H)	0.03
6	7.30 (1H, td, J = 7.5, 0.5 Hz)	7.28 (td, J = 7.4, 1.2 Hz, 1H)	0.02
7	7.45 (1H, td, J = 7.8, 1.1 Hz)	7.44 (td, J = 7.6, 1.4 Hz, 1H)	0.02
8	7.57 (1H, d, J = 7.6 Hz)	7.54 (s, 1H)	0.03
11	4.40 (1H, d, J = 9.0 Hz)	4.40 (d, J = 8.9 Hz, 1H)	0
12	2.52 (1H, m)	2.57 – 2.42 (m, 1H)	0
13	2.74 (1H, dd, J = 14.3, 5.3 Hz)	2.72 (dd, J = 14.3, 5.2 Hz, 1H)	0.02
	3.17 (1H, dd, J = 14.3, 7.8 Hz)	3.15 (dd, J = 14.3, 7.7 Hz, 1H)	0.02
14	4.93 (1H, m)	4.93 (dd, J = 7.7, 5.2 Hz, 1H)	0
19	8.29 (1H, d, J = 8.0 Hz)	8.27 (d, J = 8.2 Hz, 1H)	0.02
20	7.63 (1H, d, J = 8.0 Hz)	7.61 (d, J = 7.8 Hz, 1H)	0.02
21	7.89 (1H, m)	7.87 (m, 1H)	0.02
22	7.74 (1H, d, J = 8.0 Hz)	7.72 (d, J = 8.1 Hz, 1H)	0.02
25	8.25 (1H, s)	8.25 (s, 1H)	0
26	1.26 (3H, t, J = 7.5 Hz)	1.27 (t, J = 6.8 Hz, 6H)	0
27	1.28 (3H, t, J = 7.5 Hz)		

Table S4. Comparison of ^{13}C NMR data of our synthetic (–)-isochaetominine C (6) with Aspera chaetominine B

Position	Aspera chaetominine B [3]<br>^{13}C NMR<br>(125 MHz, Methanol-d_4)	This work<br>^{13}C NMR<br>(125 MHz, Methanol-d_4)	Δ<br>(ppm)
2	85.9	85.9	0
3	75.6	75.6	0
4	135.5	135.8	-0.3
5	125.4	125.4	0
6	126.8	126.8	0
7	131.7	131.7	0
8	116.1	116.1	0
9	142.0	141.8	0.2
10	175.5	175.4	0.1
11	71.5	71.5	0
12	30.4	30.4	0
13	35.7	35.8	-0.1
14	58.0	58.0	0
15	162.6	162.1	0.5
17	159.0	159.0	0
18	123.2	123.2	0.1
19	127.6	127.6	0
20	128.8	128.8	0
21	136.0	136.0	0
22	128.0	128.0	0
23	148.8	148.8	0
25	148.0	147.9	0.1
26	19.4	19.4	0
27	20.7	20.7	0

References

[1] Mao, Z.-Y.; Geng, H.; Zhang, T.-T.; Ruan, Y.-P.; Ye, J.-L.; Huang, P.-Q. *Org. Chem. Front.* **2016**, *3*, 24-37. doi:10.1039/c5qo00298b

Correction: Mao, Z. Y.; Mao, Z.-Y.; Geng, H.; Zhang, T.-T.; Ruan, Y.-P.; Ye, J.-L.; Huang, P.-Q. *Org. Chem. Front.* **2024**, *11*, 2693-2693. doi:10.1039/D4QO90034K

[2] Wu, J.-F.; Huang, P.-Q. *Chin. Chem. Lett.* **2020**, *31*, 61-63. doi:10.1016/j.cclet.2019.06.043

[3] Fredimoses, M.; Ai, W.; Lin, X.-P.; Zhou, X.-F.; Liao, S.-R.; Pan, L.; Liu, Y.-H. *Nat. Prod. Res.* **2025**, *39*, 566-578. doi:10.1080/14786419.2023.2275744

^{1}H NMR and ^{13}C NMR spectra of new products

^{1}H NMR spectrum of **19** and **20** (DMSO-d_6)

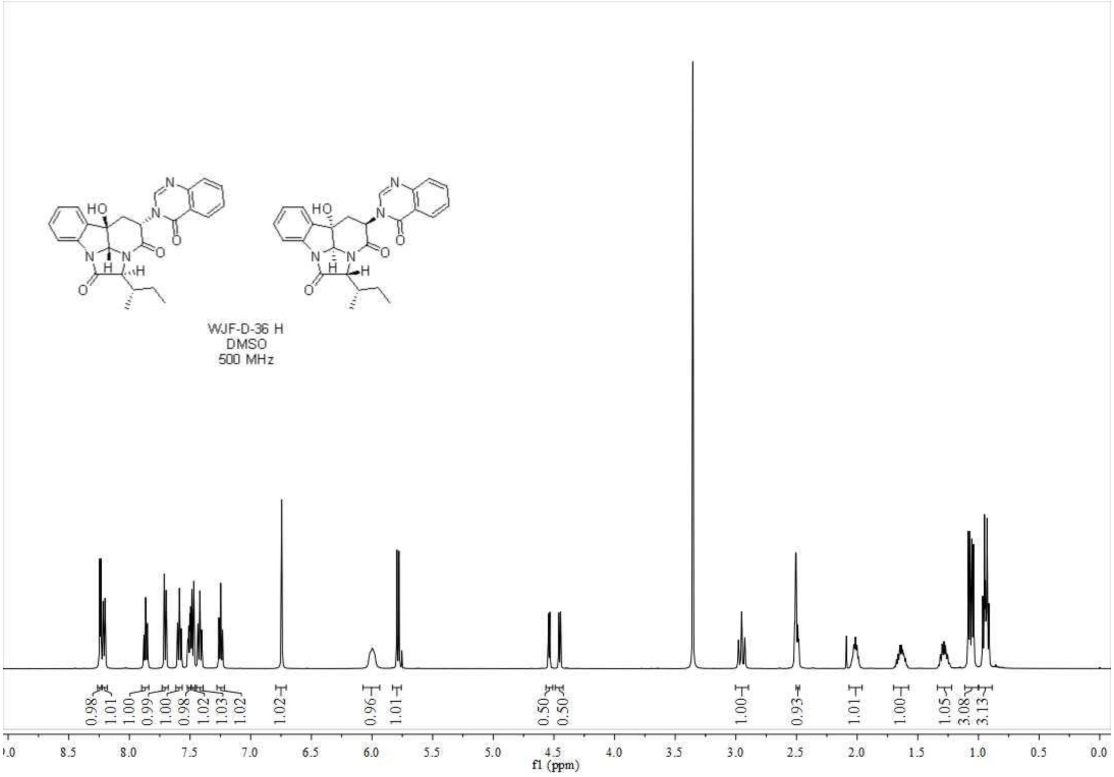

^{13}C NMR spectrum of **19** and **20** (DMSO-d_6)

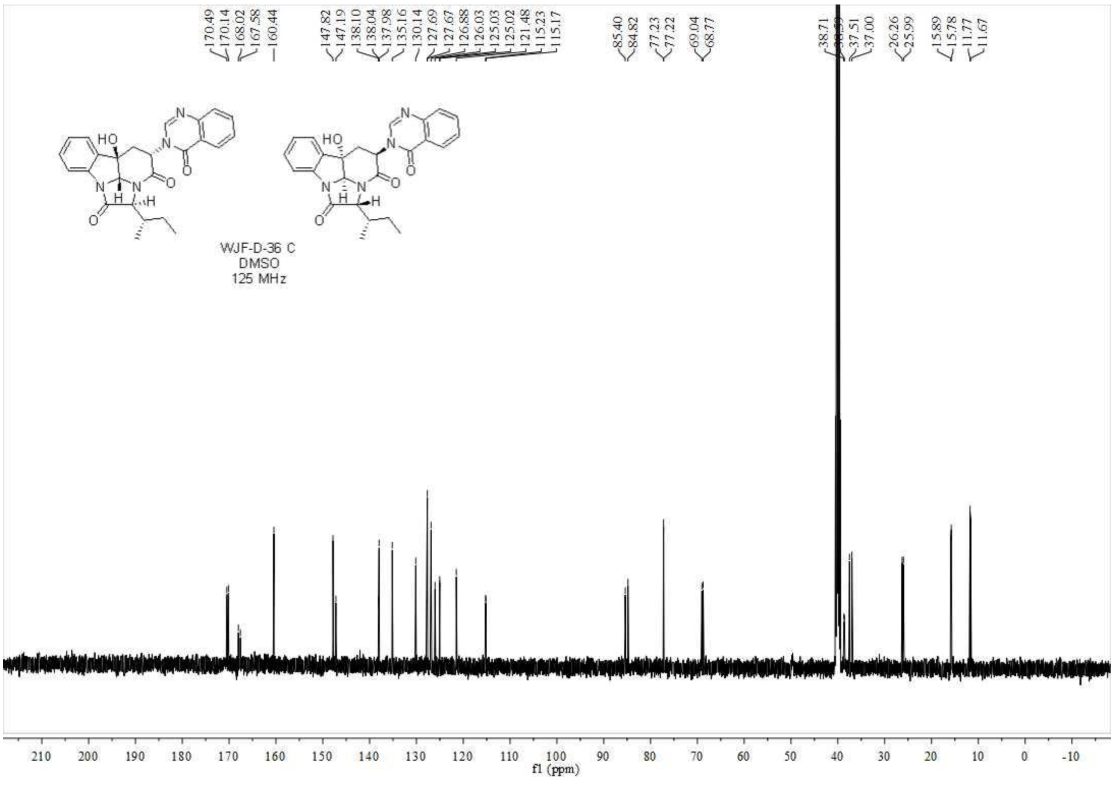

^{1}H NMR spectrum of **25** ($CDCl_3$)

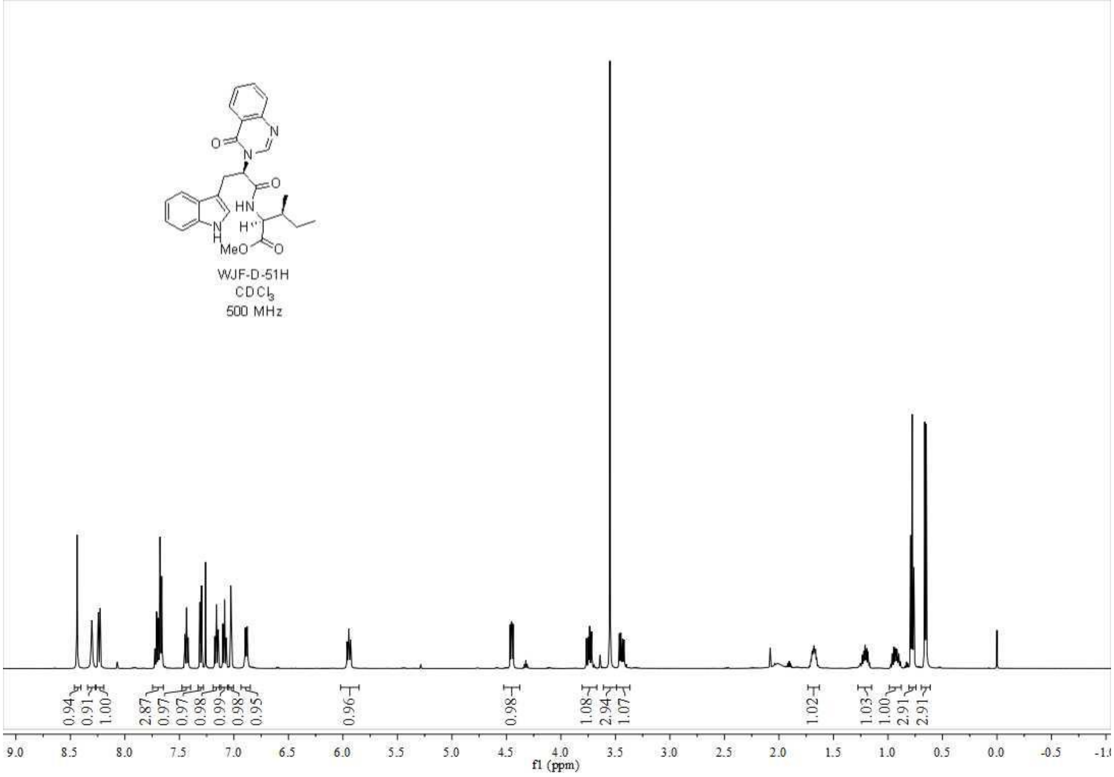

^{13}C NMR spectrum of **25** ($CDCl_3$)

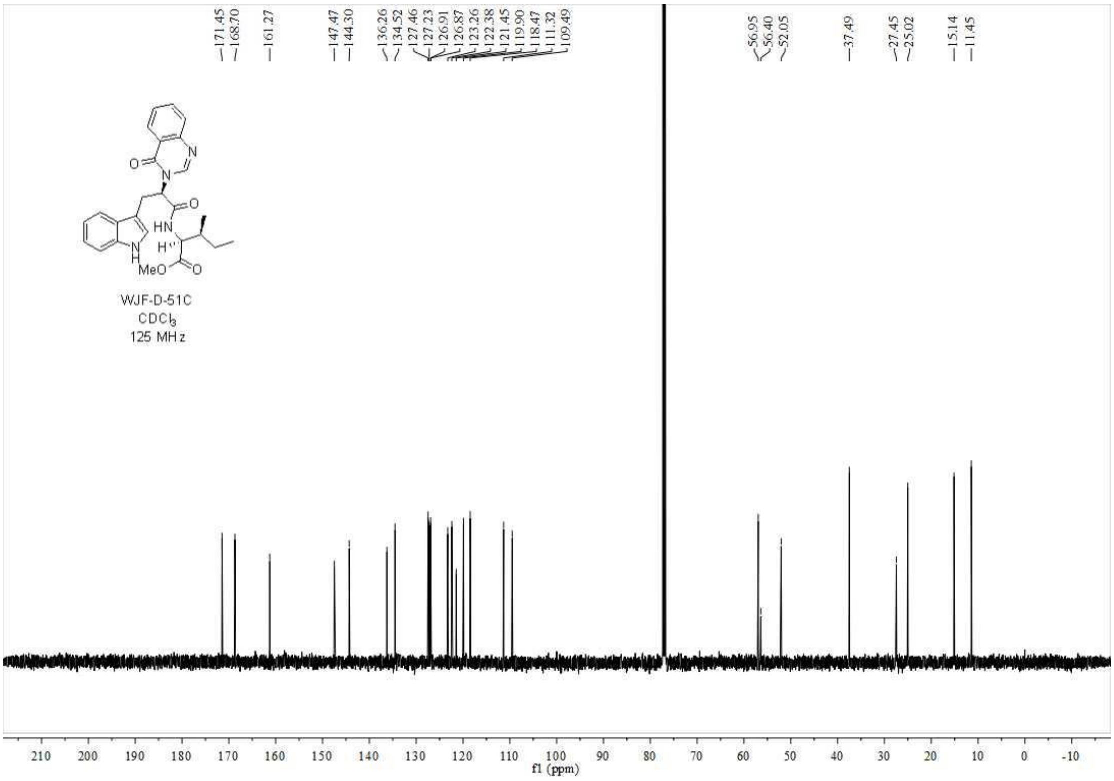

^{1}H NMR spectrum of **26** ($CDCl_3$)

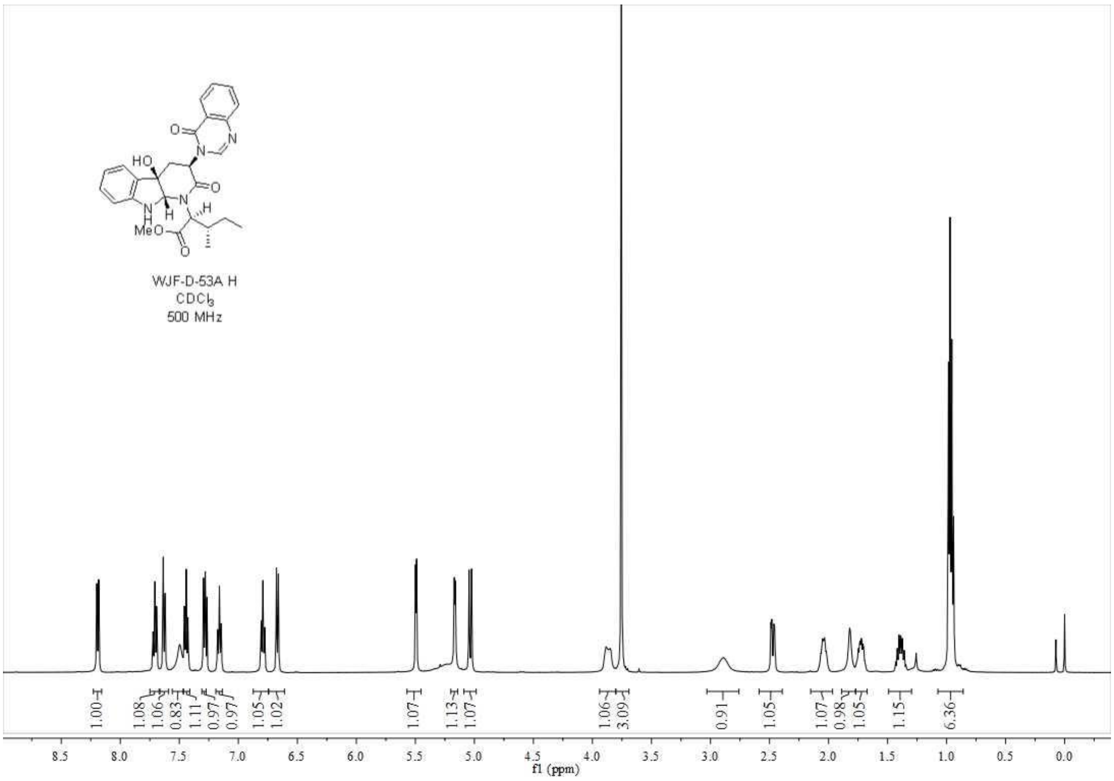

^{13}C NMR spectrum of **26** ($CDCl_3$)

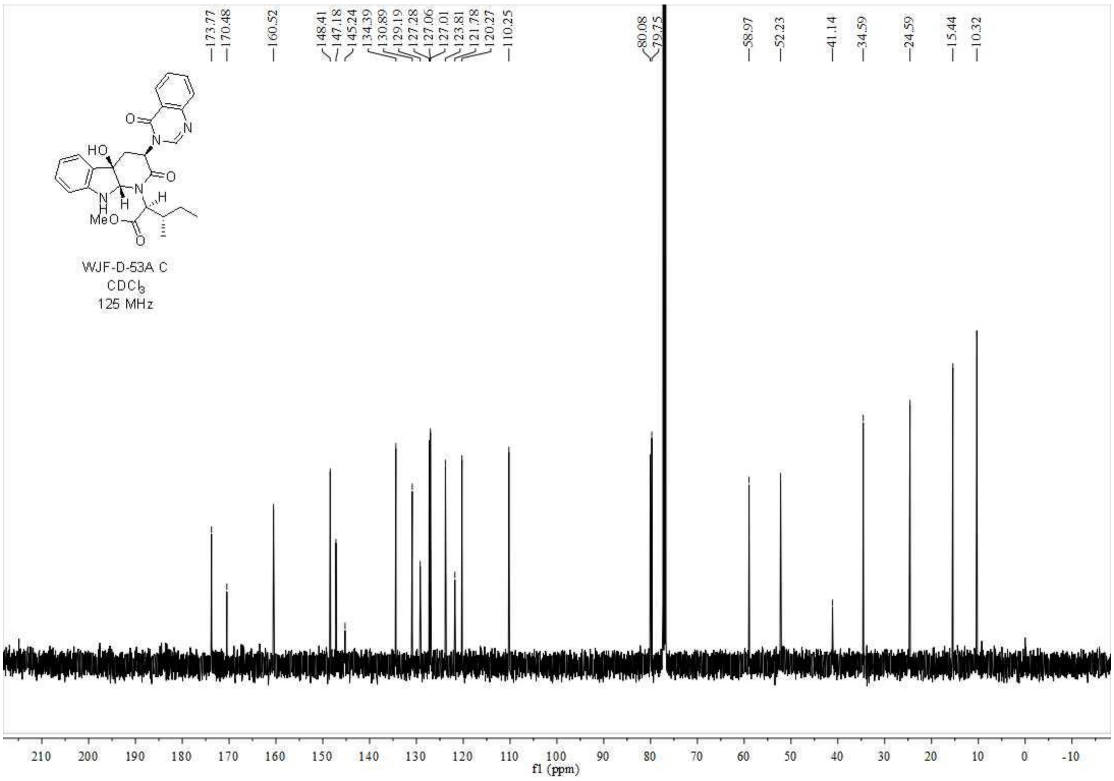

^{1}H NMR spectrum of **12** (methanol-d_4)

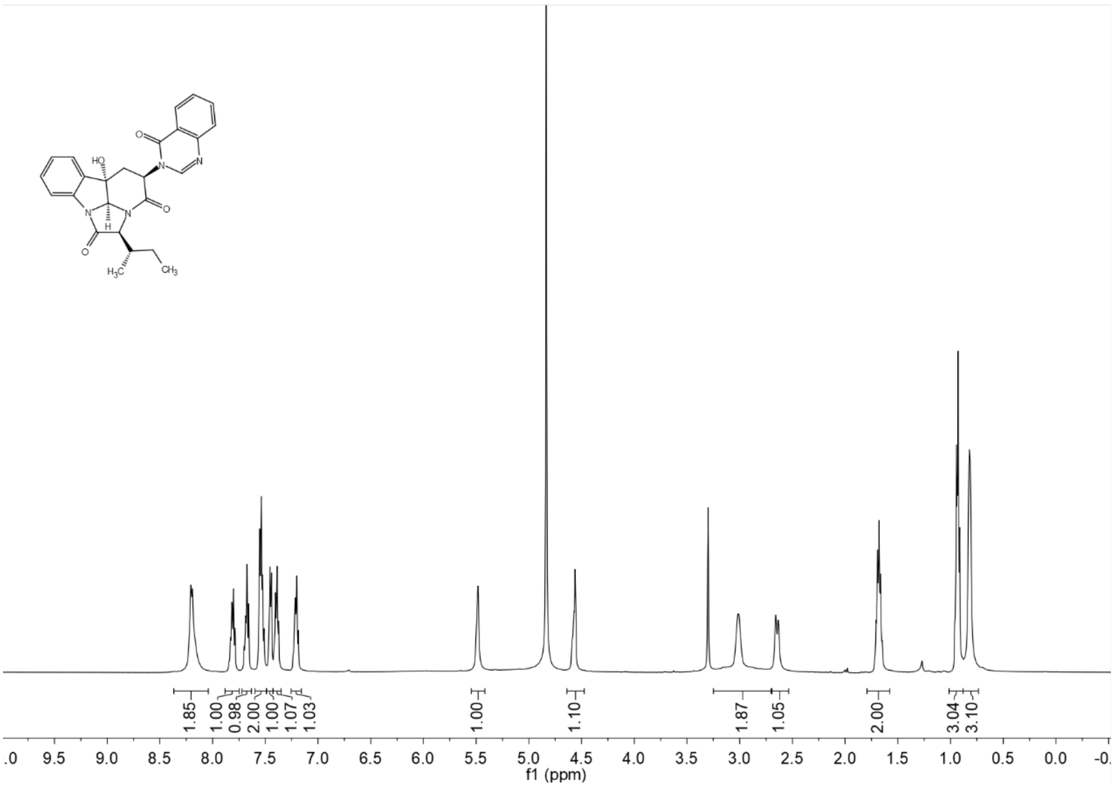

^{13}C NMR spectrum of **12** (methanol-d_4)

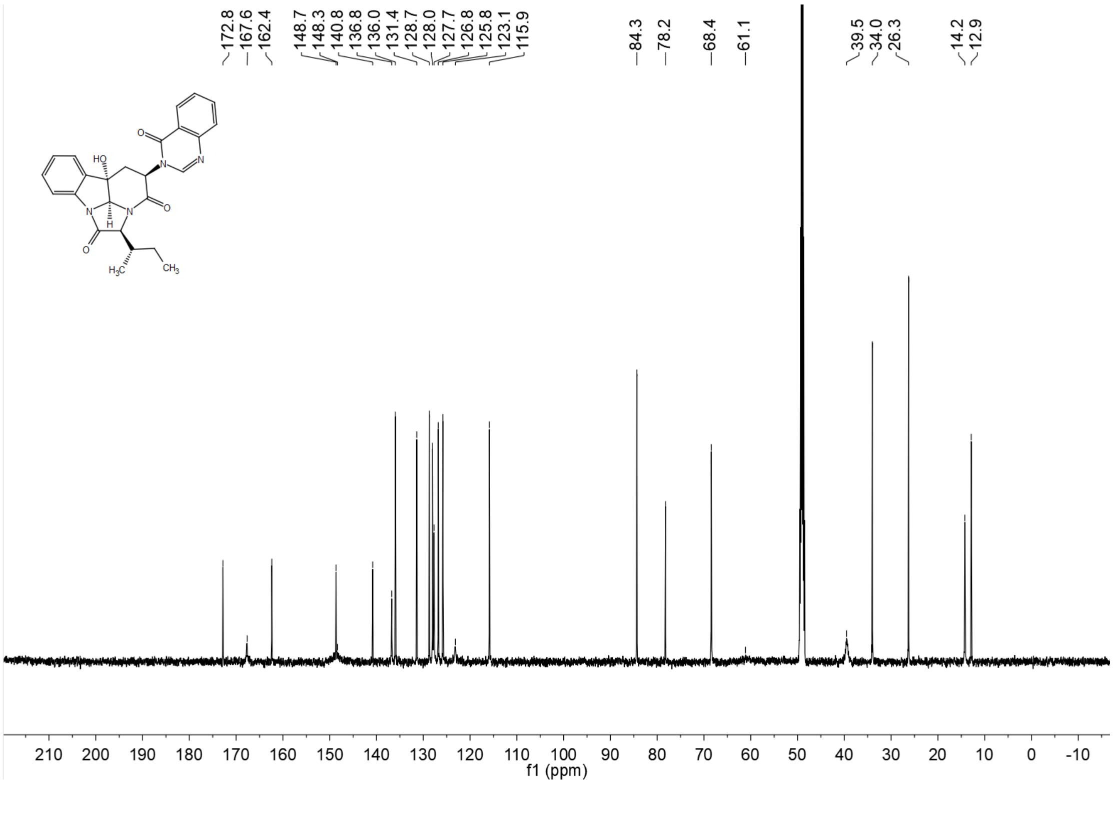

^{1}H NMR spectrum of **13** (methanol-d_4)

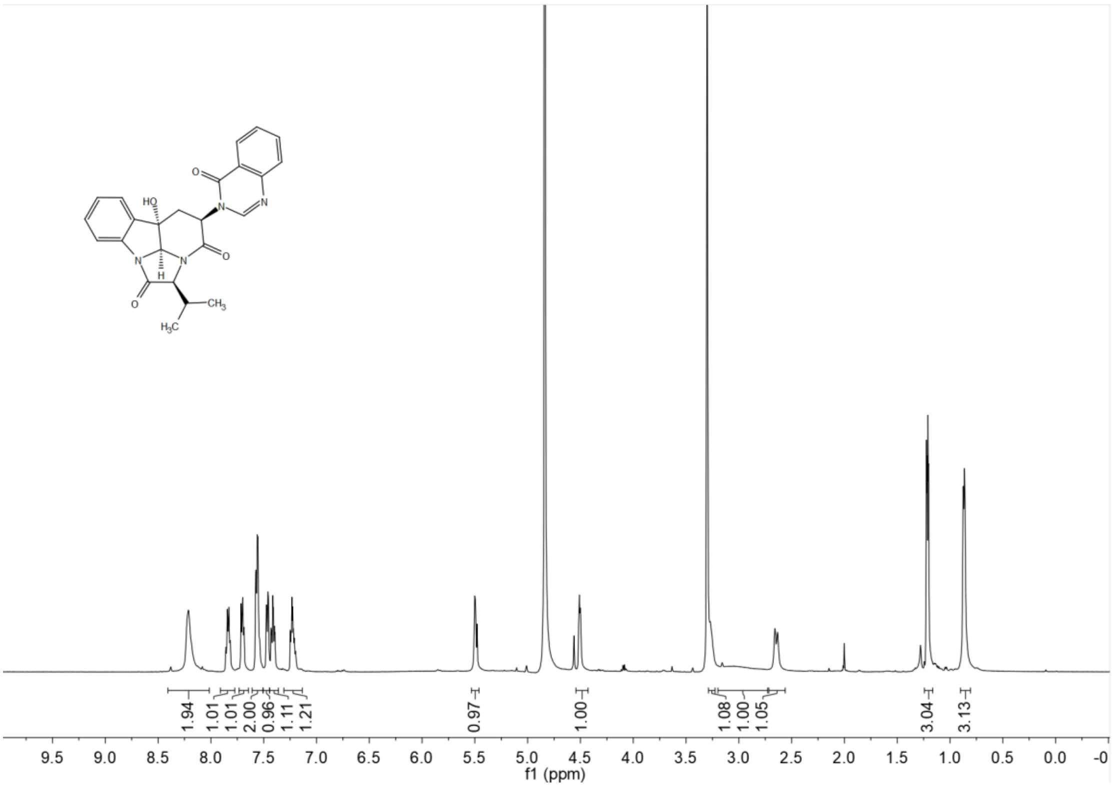

^{13}C NMR spectrum of **13** (methanol-d_4)

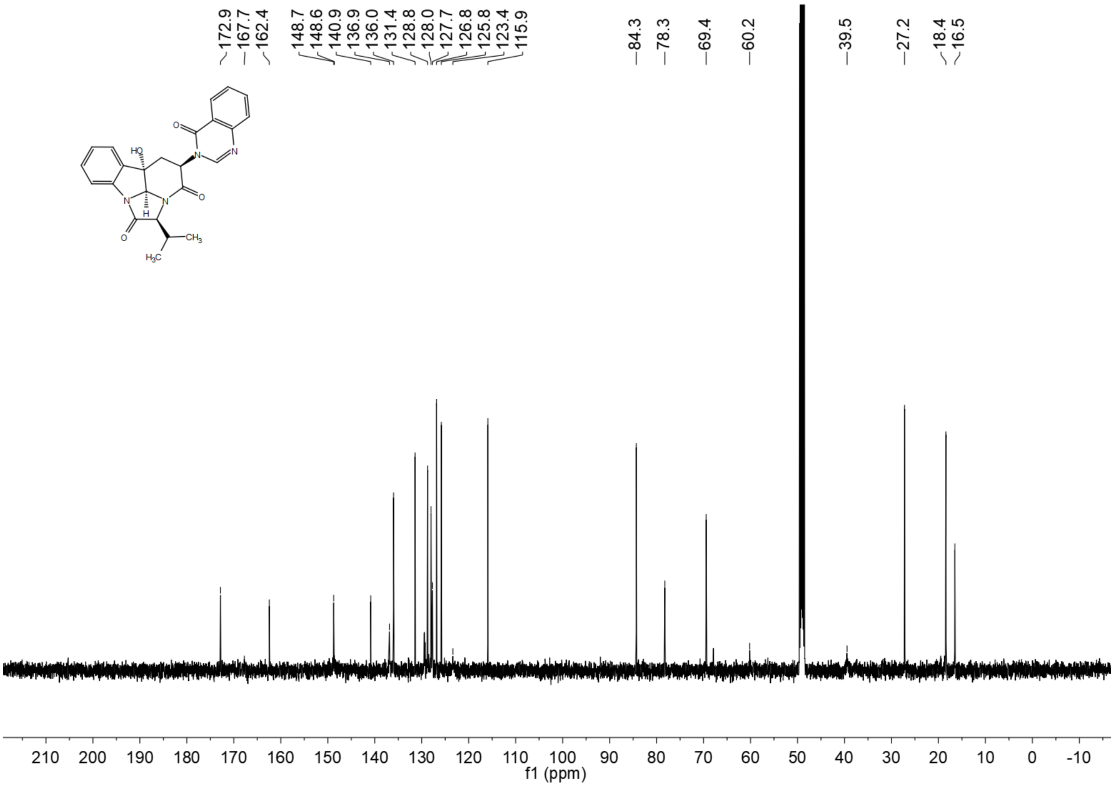

^{1}H NMR spectrum of **6** (methanol-d_4)

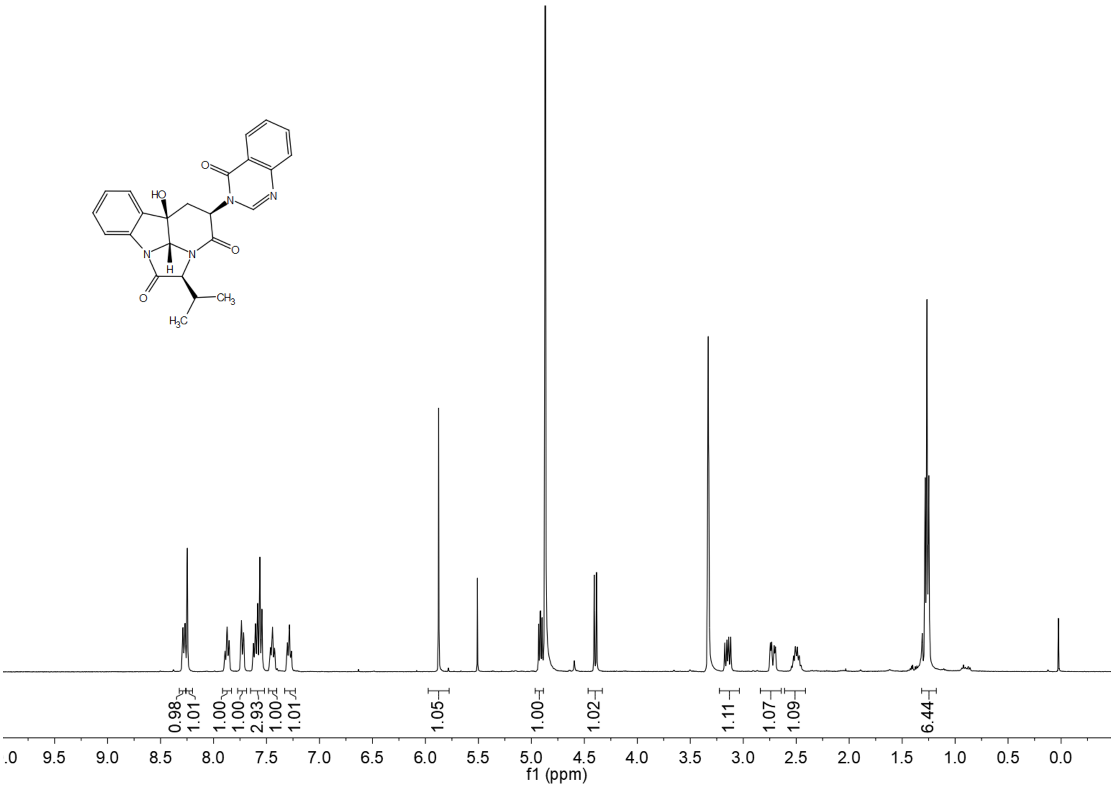

^{13}C NMR spectrum of **6** (methanol-d_4)

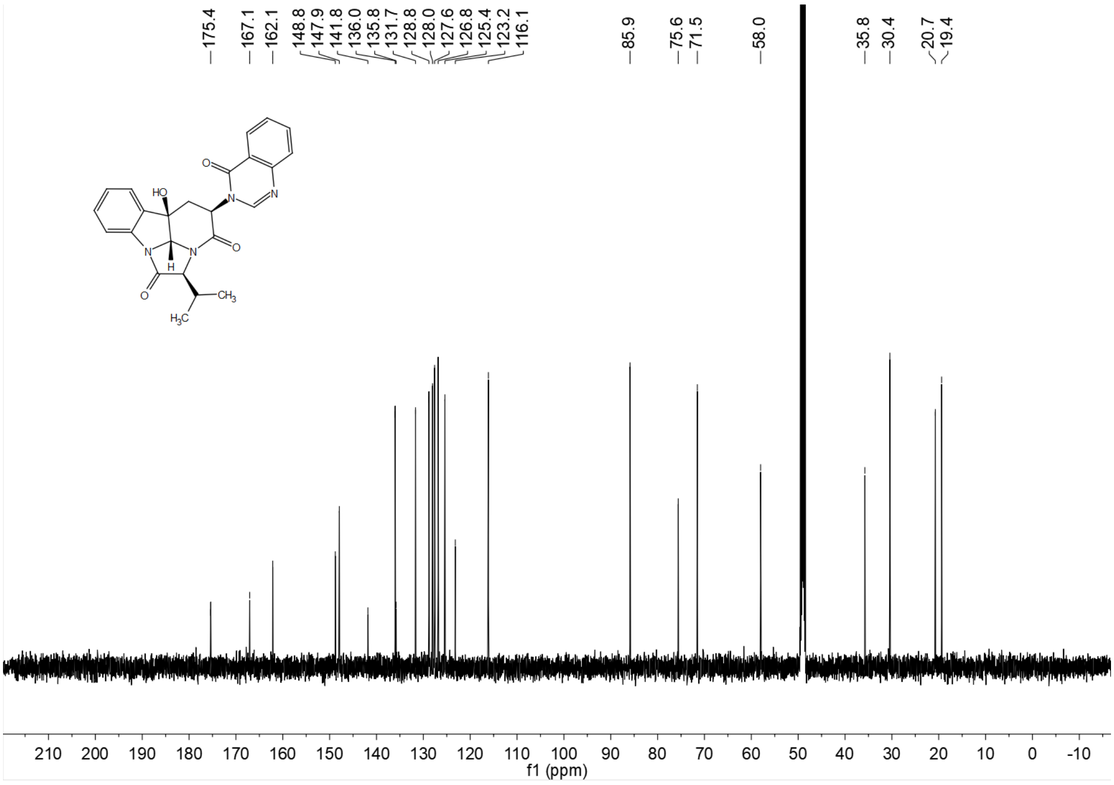

Supplement: File 1 — General methods and materials, experimental procedures, characterization data, and copies of 1H and 13C NMR spectra of compounds 19/20, 25, 26, 12, 13, and 6. [file Beilstein_J_Org_Chem-21-2072-s001.pdf]
